# Neutrophil-to-lymphocyte ratio as a prognostic biomarker in patients with peripheral artery disease: A systematic review and meta-analysis

**DOI:** 10.1177/1358863X241281699

**Published:** 2024-10-16

**Authors:** Roy B Kurniawan, Paulus P Siahaan, Pandit BT Saputra, Jannatin N Arnindita, Cornelia G Savitri, Novia N Faizah, Luqman H Andira, Mario D’Oria, J Nugroho Eko Putranto, Firas F Alkaff

**Affiliations:** 1 Faculty of Medicine, Universitas Airlangga, Surabaya, Indonesia; 2 Department of Cardiology and Vascular Medicine, Faculty of Medicine, Universitas Airlangga-Dr. Soetomo General Academic Hospital, Surabaya, Indonesia; 3 Cardiovascular Research and Innovation Center, Universitas Airlangga, Surabaya, Indonesia; 4 Division of Cardiovascular Medicine, Graduate School of Medicine, Kobe University, Kobe, Japan; 5 Division of Vascular and Endovascular Surgery, Department of Clinical Surgical and Health Sciences, University of Trieste, Trieste, Italy; 6 Division of Nephrology, Department of Internal Medicine, University of Groningen, University Medical Center Groningen, Groningen, Netherlands; 7 Department of Anatomy, Histology, and Pharmacology, Division of Pharmacology and Therapy, Faculty of Medicine, Universitas Airlangga, Surabaya, Indonesia

**Keywords:** major adverse cardiovascular events (MACE), major adverse limb events (MALE), mortality, neutrophil-to-lymphocyte ratio, peripheral artery disease (PAD), inflammation

## Abstract

**Background:**

The neutrophil-to-lymphocyte ratio (NLR) is a simple and routinely obtained parameter reflecting systemic inflammation, including in peripheral artery disease (PAD).

**Methods:**

This systematic review aimed to assess the role of NLR as a prognostic biomarker in patients with PAD. A systematic search was conducted across PubMed, ScienceDirect, Web of Science, Scopus, ProQuest, EBSCO, and Cochrane. Random-effects meta-analysis was used to pool risk ratios, sensitivity, specificity, positive predictive value (PPV), and negative predictive value (NPV). A bivariate model was used to generate summary receiver operating characteristics with the corresponding area under the curve (AUC).

**Results:**

This review included 5243 patients with PAD from nine eligible studies. High NLR corresponded to at least a twofold increased risk of all-cause mortality (ACM), major adverse limb events (MALE), and major adverse cardiovascular events (MACE). NLR’s performance was good for predicting 1-year ACM (AUC 0.71 [95% CI: 0.59–0.79], sensitivity 58.2% [95% CI: 45.3–71.0], specificity 72.6% [95% CI: 65.6–79.62], PPV 41.0% [95% CI: 31.2–50.7], NPV 82.7% [95% CI: 74.1–91.3]) and 1-year MALE (AUC 0.78 [95% CI: 0.75–0.80], sensitivity 65.4% [95% CI: 41.6–89.2], specificity 77.7% [95% CI: 71.0–84.3], PPV 53.7% [95% CI: 47.3–60.1], NPV 83.91% [95% CI: 73.2–94.6]). However, these values tended to decrease as the follow-up duration extended, except for the pooled specificities, which exhibited the opposite pattern.

**Conclusion:**

NLR emerges as a simple and cost-effective prognostic biomarker with decent performance for poor outcomes in patients with PAD **(PROSPERO Registration No.: CRD42023486607)**.

## Background

Peripheral artery disease (PAD) is an atherosclerotic manifestation predominantly affecting the lower extremities, leading to narrowed arteries and compromised blood flow. This condition often results in functional impairments with its most severe phenotype – chronic limb-threatening ischemia (CLTI).^1^ CLTI causes rest pain, wounds, and amputation, which could limit patients’ physical activities.^1,2^ Patients with PAD commonly experience significant pain in the extremities, and face a heightened risk of major adverse limb events (MALE), major adverse cardiovascular events (MACE), and increased mortality rates.^3,4^ From 1990 to 2019, there has been a substantial 72% increase in diagnosed PAD cases, estimated to have risen from 65 million to over 110 million individuals. Moreover, there has been a 13% increase in PAD prevalence per 100,000 persons during this period.^5^ Despite affecting more than 200 million individuals globally, over 50% of PAD cases are asymptomatic, with a crude 5-year death rate of 82.4 per 1000 patient-years.^6^ Geographically, PAD prevalence is observed to be higher in Africa (6.7%) compared to Europe (3.5%) and Asia (6.7%).^7^

Similar to coronary artery disease (CAD), PAD is an atherosclerotic disease which also triggers inflammatory pathways.^8^ Proatherosclerotic factors, such as hyperlipidemia and oxidative damage, can trigger systemic inflammation.^9^ This inflammation is currently regarded as the core mechanism of atherosclerotic formation.^10^ It leads to the release of pro-inflammatory cytokines, resulting in an increased neutrophil count.^9^ Concurrently, this inflammatory state is also associated with a low lymphocyte count.^11^ The neutrophil-to-lymphocyte ratio (NLR) has emerged as a superior indicator compared to individual white blood cell counts in assessing various inflammatory conditions, such as cardiovascular disease, chronic liver disease, malignancies, acute respiratory distress syndrome, and sepsis.^12–17^

Given its performance in evaluating inflammatory conditions, the NLR holds promise as a predictive biomarker in PAD, considering its underlying inflammatory nature. Previous reports have also found the relationship between both neutrophil alone and NLR with mortality and amputation among patients with PAD.^18–20^ Additionally, NLR is also a cost-effective and relatively reliable marker compared to others. However, the predictive potential of the NLR to predict poor outcomes in patients with PAD remains to be elucidated. This systematic review and meta-analysis aimed to explore the association between NLR and PAD outcomes while assessing its performance to predict those outcomes, namely all-cause mortality, MACE, and MALE among patients with PAD.

## Methods

### Study design

This systematic review and meta-analysis followed the criteria outlined in the Preferred Reporting Items for Systematic Review and Meta-analysis (PRISMA) 2020 guidelines (Table S1).^21^ Additionally, the protocol for this review was officially registered in the International Prospective Register of Systematic Reviews (PROSPERO) database (Registration No.: CRD42023486607).

### Eligibility criteria

This review included both prospective and retrospective observational studies. Potential studies were screened based on the following criteria: (1) involvement of adult patients (> 18 years old) with lower peripheral vascular disease (e.g., CLTI, or lower-extremity PAD)^22^; (2) availability of NLR data, including the number of patients with NLR above or below a specified cut-off value; (3) reporting of at least one outcome of interest in this study (all-cause mortality (ACM), MACE, and MALE); and (4) publication in the English language. Studies considered case reports and case series were excluded from this review. The outcome of MACE is defined as any events of nonfatal myocardial infarction or nonfatal stroke or cardiac-related death, whereas MALE is defined as an amputation ascribed to a vascular event.

### Literature search and study selection

A systematic search strategy was designed to identify relevant studies for inclusion in this systematic review. Multiple databases including PubMed, ScienceDirect, Web of Science, Scopus, ProQuest, EBSCO, and Cochrane were systematically searched from their inception date until January 1, 2024. The search strategy employed a combination of keywords related to (“Neutrophil-to-Lymphocyte”) OR (“Neutrophil-to-Lymphocyte Ratio”) AND (“PAD”) OR (“Peripheral artery disease”) OR (“Peripheral arterial disease”). Detailed search strategies are provided in Table S2.

**Figure 1.**
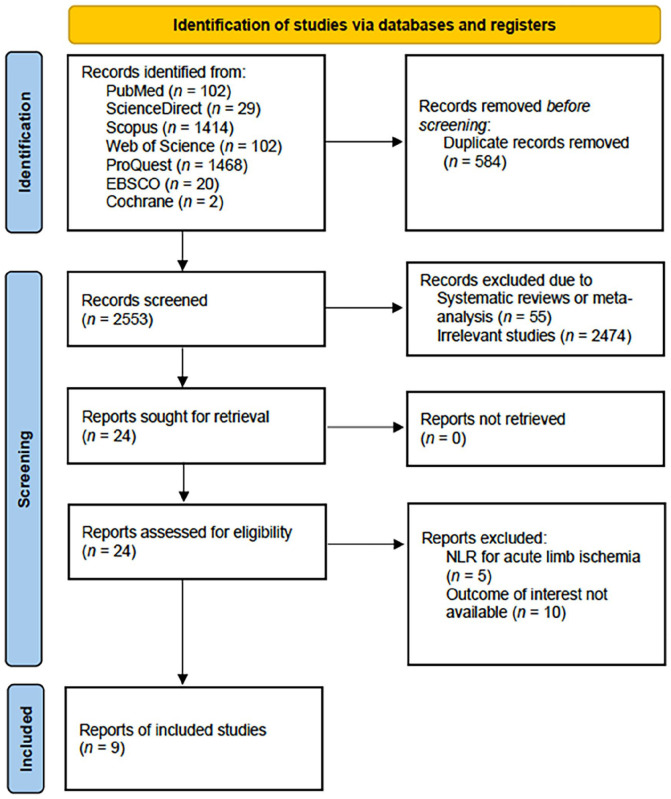
PRISMA flow diagram of the study selection process. NLR, neutrophil-to-lymphocyte ratio.

Titles and abstracts of retrieved studies were further screened for eligibility based on predefined inclusion and exclusion criteria by two authors (PPS and RBK), independently. Collected studies underwent full-text review to assess their eligibility. Any discrepancies during the screening process were discussed together with the authors. The inclusion and exclusion of studies were documented in accordance with the PRISMA flow diagram (see Figure 1).

### Quality assessment

The methodological quality of the included studies was evaluated using the Newcastle-Ottawa Scale (NOS), a widely accepted tool for assessing the quality of observational studies. At least two review authors (PPS and JNA) conducted independent assessments to determine the risk of bias in each study. The NOS assesses studies across three fundamental domains: patient selection, comparability, and outcomes.^23^ Studies were assigned scores ranging from 0 to 9 points based on their performance across these domains. Those achieving scores of 7 to 9 points were classified as high quality, indicating a low risk of bias and greater methodological robustness. Studies scoring between 4 and 6 points were categorized as moderate quality, suggesting a moderate risk of bias but still considered acceptable for inclusion. Conversely, studies with scores from 0 to 3 points were deemed low quality, indicating a higher risk of bias and potential methodological limitations. Discrepancies were resolved through discussion or consultation with a senior author when needed.

### Data extraction

Relevant data from the selected studies were systematically extracted and tabulated by at least two authors (PPS and RBK), independently. This included details such as first author’s name, publication year, study period, study design, country of origin, population demographics, percentages of patients with comorbidities (hypertension, CAD, diabetes mellitus [DM], dyslipidemia), and sample sizes. Furthermore, outcomes of interest, including ACM, MACE, and MALE, were extracted alongside NLR cut-off values, predictive performance raw values (true positive [TP], false negative [FN], true negative [TN], false positive [FP]), and respective follow-up durations for each outcome. Whenever those metrics were not directly obtained, we followed the University of Oxford Centre of Evidence-Based Medicine (CEBM) guideline to generate those numbers.^24^ Any disagreements during the data extraction process were discussed together with the senior authors.

### Statistical analysis

The analysis was conducted using R software version 4.2.2 (Posit PBC, Boston, MA, USA) and Review Manager version 5.4 (Cochrane Collaboration, London, UK). A random-effects model meta-analysis was employed to account for the heterogeneity nature of the included studies. In this analysis, we estimated the risk ratio (RR) and evaluated the performance of the NLR in predicting outcomes among patients with PAD. Predictive performance metrics included sensitivity, specificity, positive predictive value (PPV), negative predictive value (NPV), and the area under the curve (AUC) in predicting outcomes among patients with PAD. Pooled sensitivity, specificity, PPV, and NPV were estimated using the univariate model while generating summary receiver operating characteristic (ROC) curves and corresponding AUC values using the bivariate model. To assess heterogeneity, Higgins’ *I*^2^ values categorized its levels as negligible (0–25%), low (25–50%), moderate (50–75%), or high (> 75%). Exploration of potential sources of heterogeneity involved planned subgroup analyses considering several factors like study design, NLR cut-off, and the country of origin of the study population. Though the prevalence of outcomes in each study was different, we did not separately investigate the observed heterogeneities in PPV and NPV as these metrics are influenced by the outcomes’ prevalence in each of the studies. Additionally, detection of outliers, Baujat plot visualization, or leave-one-out sensitivity (LOOS) analysis were performed to aid in explaining the observed heterogeneity. We considered data as an outlier when its confidence interval laid outside the pooled estimate. Further analyses such as metaregression and evaluation for publication bias using Egger’s test and Begg’s funnel plot were considered if there were at least 10 studies included in any pooled analysis. Statistical significance was established at a *p*-value less than 0.05 for all analyses conducted.

## Results

### Study inclusion and quality assessment

Upon completing the database search, a total of 3137 studies were initially identified. Following the removal of 584 duplicates, the articles underwent screening based on the predefined inclusion criteria. Ten studies were subsequently excluded due to not reporting the outcome of interest^18,25–33^ and five studies were excluded because the study was done in a population of patients with acute limb ischemia.^34–38^ A total of nine studies met the criteria for inclusion in this review.^39–43^ Comprehensive details regarding the study selection process can be found in the PRISMA flow diagram (Figure 1). The selected studies were further evaluated using the NOS critical appraisal checklist to ensure methodological rigor. Three studies were identified as high quality,^39,44,45^ and the remaining studies qualified as moderate quality (Table S3).

### Study characteristics

A total of nine studies involved 5243 subjects with PAD. All these studies were either retrospective or prospective cohort studies and were published between 2010 and 2022 (Table 1). The mean age of patients was over 64 years old and most patients were men (Table 1). Of the identified studies, nine reported PAD severities ranging from Rutherford grades II to VI. The studies also documented common comorbidities such as hypertension (HT), CAD, DM, and dyslipidemia (DLD). Detailed characteristics of the studies are presented in Tables 1 and 2.

**Table 1.** Characteristics of included studies.

Reference	Study design	Country	Population	Age (years), mean ± SD	Male sex (%)	PAD classification	Comorbidities (%)			
							HT	CAD	DM	DLD
Adler et al., 2022^42^	Cohort prospective	United States	Patients with LEAD receiving open operative management	71.98 ± 10.09	64.5	Rutherford grade III–VI	53	14	17	n/a
Bath et al., 2020^40^	Cohort prospective	United States	Patients with PAD receiving any operative management	69 ± 11	61.7	Fontaine stage II–IV	n/a	50	41	n/a
Erturk et al., 2014^43^	Cohort retrospective	Turkey	Patients with PAD receiving any operative or nonoperative management	64 ± 10	81.3	Rutherford grade IV–VI	61	41	42	62
González-Fajardo et al., 2014^45^	Cohort prospective	Spain	Patients with CLTI receiving any operative management	73.0 ± 13.8	83.0	Rutherford grade IV–VI	63	23	46	37
King et al., 2021^41^	Cohort retrospective	United States	Patients with PAD receiving endovascular operative management	71.7 ± 12.8	55.5	n/a	91	50	57	62
Luo et al., 2015^58^	Cohort prospective	China	Patients with CLTI receiving nonoperative management	71.98 ± 10.09	70.9	Rutherford grade IV–V	53	14	17	n/a
Russu et al., 2022^44^	Cohort retrospective	Romania	Patients with PAD receiving open operative management	72.95 ± 11.45	74.1	Rutherford grade II–V	83	35	49	59
Spark et al., 2010^39^	Cohort prospective	United States	Patients with CLTI receiving any operative or nonoperative management	69.72 ± 8.35	n/a	n/a	55	6	29	n/a
Su and Lui, 2021^46^	Cohort retrospective	Taiwan	Patients with CLTI receiving endovascular operative management	74 ± 11.5	51.8	Rutherford grade IV–VI	66	38	69	17

CAD, coronary artery disease; CLTI, chronic limb-threatening ischemia; DLD, dyslipidemia; DM, diabetes mellitus; HT, hypertension; LEAD, lower-extremity artery disease; n/a, not available; PAD, peripheral artery disease.

**Table 2.** Individual outcomes of included studies.

Reference	Sample size (*N*)	Cut-off (ng/L)	Outcome	PPV	NPV	Sensitivity	Specificity	AUC^a^	YJI
Adler et al., 2022^42^	199	4.6 (pretreatment)	1-year ACM	0.40	0.68	0.46	0.62	0.563	0.08
	129		5-year ACM	0.80	0.44	0.46	0.78	0.690	0.24
	254		30-day ACM	0.21	0.78	0.38	0.59	n/e	–0.03
Bath et al., 2020^40^	2701	5.96 (posttreatment)	In-hospital ACM	0.01	1.00	0.85	0.35	0.693	0.20
	2701		In-hospital MALE	0.08	0.97	0.85	0.36	0.698	0.21
	2701		In-hospital MACE	0.04	0.97	0.72	0.35	0.568	0.07
Erturk et al., 2014^43^	458	3 (no treatment / pretreatment)	2-year ACM	0.33	0.90	0.57	0.77	0.736	0.34
González-Fajardo et al., 2014^45^	561	5 (pretreatment)	1-year ACM	0.29	0.88	0.38	0.83	0.691	0.21
			1-year MALE	0.56	0.72	0.33	0.87	0.702	0.20
			2-year ACM	0.34	0.82	0.32	0.83	0.655	0.15
			2-year MALE	0.60	0.64	0.29	0.86	0.667	0.16
			3-year ACM	0.42	0.78	0.32	0.84	0.665	0.17
			3-year MALE	0.66	0.59	0.29	0.87	0.678	0.16
			4-year ACM	0.46	0.71	0.28	0.84	0.637	0.12
			4-year MALE	0.70	0.54	0.27	0.88	0.676	0.15
			5-year ACM	0.49	0.67	0.27	0.84	0.629	0.11
			5-year MALE	0.74	0.50	0.27	0.89	0.688	0.16
King et al., 2021^41^	488	4 (pretreatment)	1-year ACM	0.41	0.82	0.59	0.69	0.693	0.28
			1-year MALE	0.56	0.79	0.63	0.74	0.746	0.37
			2-year ACM	0.68	0.55	0.48	0.73	0.662	0.22
			2-year MALE	0.78	0.52	0.50	0.79	n/e	0.29
			3-year ACM	0.83	0.36	0.45	0.77	0.677	0.22
			3-year MALE	0.89	0.34	0.45	0.83	0.726	0.28
			4-year ACM	0.92	0.17	0.41	0.78	0.663	0.18
			4-year MALE	0.95	0.16	0.41	0.82	0.698	0.24
Luo et al., 2015^58^	172	3.8 (posttreatment)	3-year MALE	0.57	0.83	0.71	0.72	0.776	0.43
Russu et al., 2022^44^	224	3.95 (pretreatment)	1-year ACM	0.27	0.97	0.85	0.69	0.839	0.54
			1-year MALE	0.42	0.97	0.90	0.73	0.881	0.63
Spark et al., 2010^39^	149	5.25 (no treatment / pretreatment)	1-year ACM	0.58	0.71	0.61	0.68	0.698	0.29
Su and Liu, 2021^46^	195	8 (pretreatment)	1-year ACM	0.54	0.86	0.62	0.82	0.792	0.44
	236		1-year MALE	0.58	0.87	0.76	0.75	0.818	0.51
	236		1-year MACE	0.30	0.87	0.49	0.75	0.682	0.24

a Estimated AUC using signal detection theory by Mueller and Zhang, 2005.^59^

ACM, all-cause mortality; AUC, area under the curve; MACE, major adverse cardiovascular events; MALE, major adverse limb events; n/e, not estimable; NPV, negative predictive value; PPV, positive predictive value; YJI, Youden’s J index.

### All-cause mortality (ACM)

#### High NLR and risk of ACM

**Figure 2.**
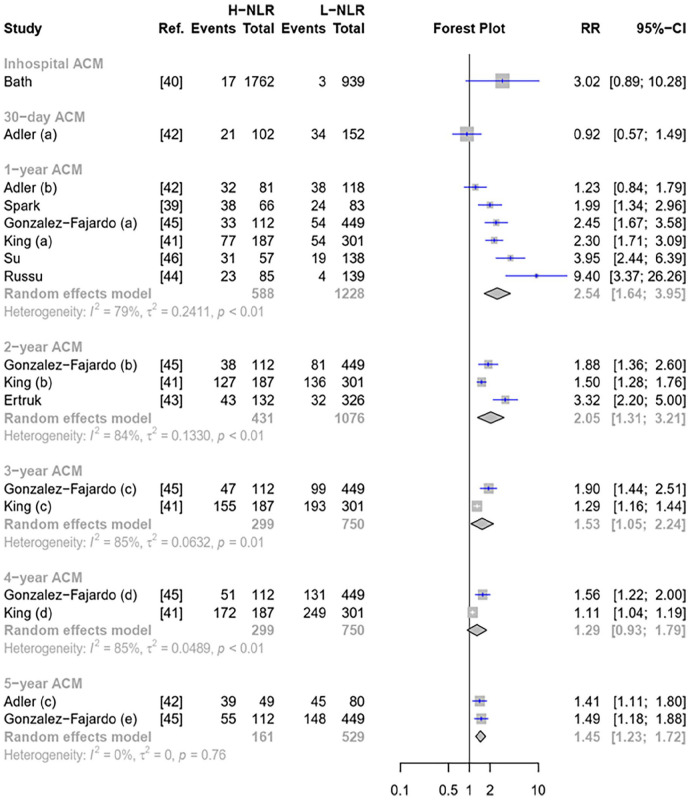
Forest plot of pooled NLR to predict ACM. ACM, all-cause mortality; H-NLR, high neutrophil-to-lymphocyte ratio; L-NLR, low neutrophil-to-lymphocyte ratio; RR, risk ratio.

A total of eight studies reported ACM outcome with varying follow-up durations, ranging from in-hospital to 5 years (Figure 2). Bath et al. was the only study that reported in-hospital mortality and only Adler et al. reported the data for 30-day ACM.^40,42^ Regardless of follow-up durations, the high NLR significantly doubled (RR 1.81 [95% CI: 1.48–2.21]) the risk of ACM among patients with PAD (Figure 2). The risk was higher when the NLR was used to predict 1-year ACM (RR 2.54 [95% CI: 1.64–3.95]), and this risk decreased as the follow-up duration extended (Figure 2).

Significant heterogeneity was observed across ACM (Figure 2). Subgroup analysis was performed on 1-year ACM outcome (Figures S1). However, a significant reduction in heterogeneity was only observed among studies with an NLR cut-off of ‘5 or more’ (Figure S1). Although outliers were not detected in both 1- and 2-year ACM (Figure 1), the Baujat plot indicated that the studies by Adler et al. and Russu et al.^42,44^ could be the sources of heterogeneity in 1-year ACM (Figure S1). It was supported by LOOS analysis (Figures S3), which showed a heterogeneity reduction after omitting Adler et al.^42^ For 2-year ACM analysis, the LOOS analysis found that Erturk et al. was the source of heterogeneity^43^ (Figure S1).

#### Performance of NLR to predict the ACM

Pooled sensitivity of NLR for predicting ACM ranged from 58.15% [95% CI: 45.34–70.97] (1-year ACM) to 36.25% [95% CI: 17.33–55.17] (5-year ACM) (Figure S2). The sensitivity of NLR to predict ACM decreased as the ACM follow-up extended. Bath et al. added that sensitivity of NLR reached 85% for predicting in-hospital ACM^40^ (Figure S2). However, significant heterogeneities were observed across analyses, despite our subgroup and outlier detection analyses. For 1-year ACM sensitivity, the heterogeneity reduction was only found in studies from North America (Figure S3). Baujat plot and LOOS analysis indicated that González-Fajardo et al. and Russu et al. might be contributors to the heterogeneity in this timeframe^44,45^ (Figure S3). The LOOS analysis (Figure S3) observed that González-Fajardo et al. was the source of heterogeneity in the 2-year ACM outcome.^45^

Pooled specificity of NLR for predicting ACM was generally > 70% and showed a positive trend as the follow-up period extended (Figure S4). Subgroup analysis partially explained the heterogeneity in the 1-year analysis, which were studies from the North American population or with NLR cut-off of three to four (Figure S5). González-Fajardo et al. was considered an outlier (Figure S4), though its omission slightly reduced the heterogeneity^45^ (Figure S5). In the 2-year analysis, no outliers were detected (Figure S4) and LOOS analysis (Figure S5) did not explain the observed heterogeneity.

Prevalence of ACM was pooled to aid assessing the pooled PPV and NPV. The prevalence of ACM in the high NLR group ranges from 41% to 69% (Figure S6). For 1-year ACM, the pooled PPV was 40.96% [95% CI: 31.22–50.70], and this value increases as the follow-up duration extends (Figure S7). Conversely, the pooled NPV of 1-year ACM was 82.67% (95% CI: 74.05–91.30), and this value decreases as the follow-up duration extends (Figure S8).

**Figure 3.**
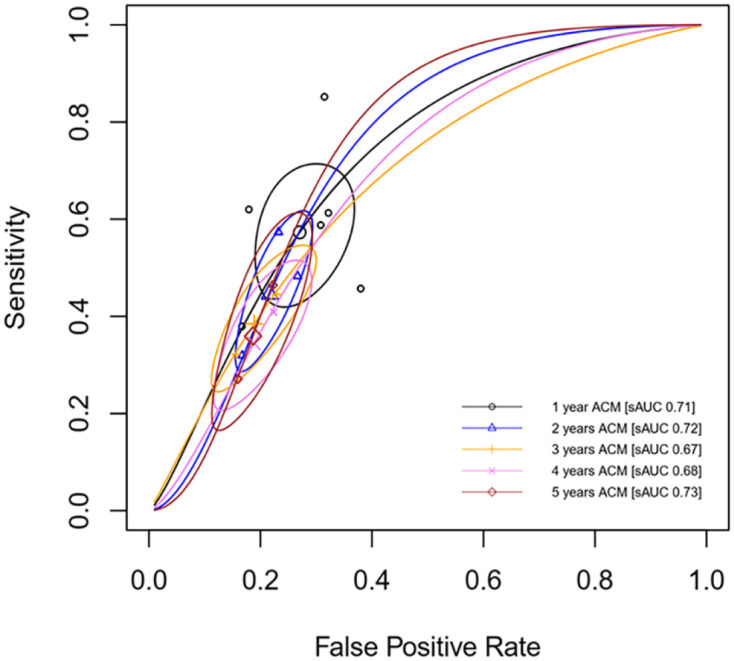
Summary ROC of NLR to predict ACM in patients with PAD. ACM, all-cause mortality; AUC, area under the curve; NLR, neutrophil-to-lymphocyte ratio; PAD, peripheral artery disease; ROC, receiver operating characteristic.

In addition, the summary ROC curve of NLR for predicting ACM found that pooled AUCs across follow-up periods were roughly similar, ranging from 0.68 to 0.73 (Figure 3). The NLR performed better in predicting 1- and 2-year ACM, which was 0.71 [95% CI: 0.59–0.79] and 0.72 [95% CI: 0.52–0.79], respectively (Table 3 and Figure 3).

**Table 3.** Pooled sensitivity, specificity, positive predictive value, negative predictive value, and area under the curve of the neutrophil-to-lymphocyte ratio to predict all-cause mortality and major adverse limb events.

Outcome	Included studies (*n*)	Included patients (*n*)		Pooled estimates				
			Prevalence of outcome (high vs low NLR)	NPV [95% CI] (%)	PPV [95% CI] (%)	Sensitivity [95% CI] (%)	Specificity [95% CI] (%)	AUC [95% CI]
**All-cause mortality**								
1-year	6	427	40.96 vs 17.33	82.67 [74.05–91.30]	40.96 [31.22–50.70]	58.15 [45.34–70.97]	72.63 [65.64–79.62]	0.71 [0.59–0.79]
2-year	3	457	44.93 vs 24.24	75.76 [54.91–96.61]	44.93 [22.15–67.72]	45.58 [31.35–59.80]	78.11 [72.38–83.85]	0.72 [0.52–0.79]
3-year	2	494	62.60 vs 43.04	56.96 [15.73–98.19]	62.60 [22.50–100.00]	38.68 [26.60–50.77]	81.39 [74.46–88.33]	0.67 [0.38–0.81]
4-year	2	603	68.95 vs 55.95	44.05 [0.00–96.53	68.95 [23.44–100]	34.65 [22.08–47.22]	82.54 [77.45–87.62]	0.68 [0.32–0.83]
5-year	2	287	64.17 vs 44.05	55.95 [33.15–78.74]	64.17 [34.30–94.04	36.25 [17.33–55.17]	83.52 [79.90–87.14]	0.73 [0.30–0.84]
**Major adverse limb events**								
1-year	4	471	53.7 vs 16.09	83.91 [73.22–69.85]	53.70 [47.33–60.07]	65.39 [41.63–89.15]	77.65 [70.98–84.31]	0.78 [0.75–0.80]
2-year	2	520	69.29 vs 42.00	58.00 [46.16–69.85]	69.29 [51.41–87.16]	39.73 [19.23–60.22]	83.15 [76.07–90.23]	0.75 [0.40 –0.84]
3-year	3	683	71.07 vs 41.69	58.31 [30.61–86.02]	71.07 [52.18–89.96]	47.96 [24.09–71.69]	81.23 [72.29–90.17]	0.77 [0.58–0.83]
4-year	2	716	82.47 vs 65.26	34.74 [0.00–72.26]	82.47 [57.97–100.00]	34.27 [20.84–47.69]	86.98 [83.37–90.59]	0.74 [0.31–0.86]

### Major adverse cardiovascular event (MACE)

Two studies investigated MACE as their outcome. Bath et al. found a nonsignificant increase in in-hospital MACE risk, whereas Su and Liu found that patients with high NLR doubled their risk of experiencing MACE in the first year^40,46^ (Figure S9). The sensitivity of NLR was better at predicting in-hospital than 1-year MACE (72% vs 49%), whereas its specificity was more accurate in predicting 1-year than in-hospital MACE. Details regarding individual PPV, NPV, and AUC of this outcome are presented in Table 2.

### Major adverse limb event (MALE)

#### High NLR and risk of MALE

**Figure 4.**
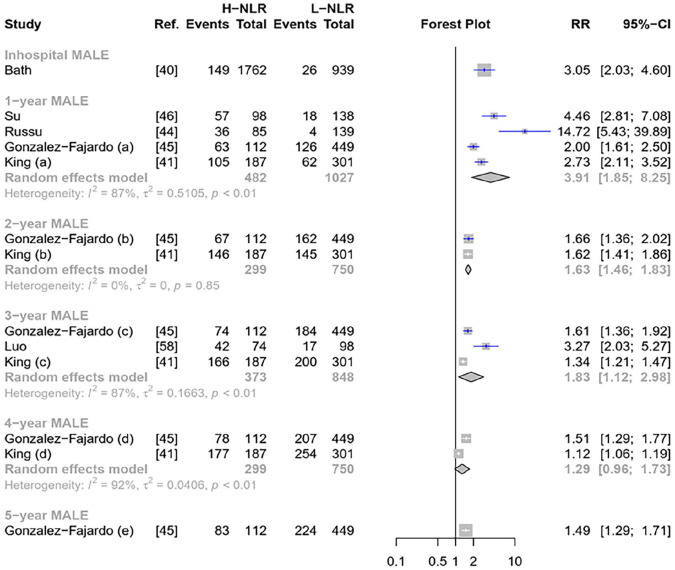
Forest plot of pooled NLR to predict MALE. H-NLR, high neutrophil-to-lymphocyte ratio; L-NLR, low neutrophil-to-lymphocyte ratio; MALE, major adverse limb event; RR, risk ratio.

Six studies investigated MALE outcome (Figure 4). Regardless of follow-up durations, high NLR increased the risk of MALE, with a better association with 1-year MALE (Figure 4). Subgroup analysis for 1-year MALE did not significantly decrease in heterogeneity (Figure S9). There were no outliers in 1-year and 3-year MALE (Figure 4). Russu et al. might contribute to the heterogeneity in the 1-year MALE outcome^44^ (Figure S10). However, LOOS analysis failed to explain those heterogeneities (Figure S10).

#### Performance of NLR to predict MALE

Pooled sensitivity of high NLR to predict 1-year MALE was best (65.39% [95% CI: 41.63–89.15]) compared to other timeframes (Figure S11). However, the sensitivity gradually decreased over the follow-up period. For 1-year MALE, subgroup analysis failed to explain heterogeneity (Figure S12). González-Fajardo et al. was considered an outlier (Figure S11), supported by Baujat plot analysis (Figure S12), yet its omission did not reduce heterogeneity^45^ (Figure S12). For 3-year MALE, no outliers were detected (Figure S11) though LOOS analysis did not reduce heterogeneity (Figure S12).

The pooled specificity of NLR was generally above 77% in predicting MALE (Figure S13). The specificity improved as the follow-up period extended. Subgroup analysis in the 1-year MALE observed that González-Fajardo et al. was the source of heterogeneity (Figure S14). However, in 1- and 3-year MALE, LOOS analyses did not find a significant reduction in heterogeneities^45^ (Figure S14).

As the prevalence of MALE among patients with high NLR was higher as the follow-up duration increased (Figure S15), the value of PPV was 53.70% (95% CI: 47.33–60.07) for 1-year follow-up and 82.47% (95% CI: 57.97–100.00) for 4-year follow-up (Figure S16). The opposite pattern was observed in NPV, as the value was lower when the follow-up duration and the prevalence of outcomes increased (Figure S17).

**Figure 5.**
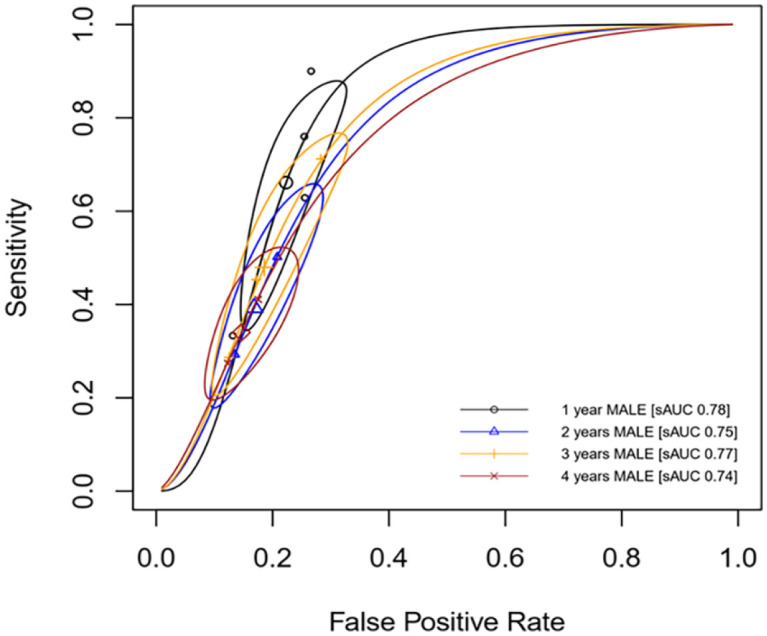
Summary ROC curve of NLR to predict MALE in patients with PAD. AUC, area under the curve; MALE, major adverse limb event; NLR, neutrophil-to-lymphocyte ratio; PAD, peripheral artery disease; ROC, receiver operating characteristic.

In addition, the best AUC for predicting MALE was for 1-year MALE (AUC 0.78 [95% CI: 0.75–0.80]) though all AUC were more than 0.70 (Table 3). Further details on the predictive performance of NLR to predict MALE outcomes are presented in Table 3 and Figure 5.

### Publication bias analysis

As there was no single analysis in ACM and MALE outcome which included at least 10 studies, publication bias analysis by Begg’s funnel plot and Egger’s regression test were not performed.

## Discussion

The NLR is one of the most feasible, affordable, and widely used inflammatory markers. It is also proposed as a potential inflammatory marker in atherosclerosis disease as the main pathophysiology of atherosclerosis involves inflammation of the vascular wall.^47^ NLR is also associated with the degree of vascular wall inflammation.^47^ Our systematic review aimed to explore the association between NLR and poor outcomes of patients with PAD and assess the predictive performance of NLR to predict those outcomes. We noted that there were significant associations between high NLR and ACM, MACE, and MALE (Figures 2 and 4). We further found that NLR exhibited a good performance in predicting those outcomes with the AUC of the summary ROC curve exceeding 0.70 (Table 3).

A previous meta-analysis reported that high NLR was associated with a 1.6-fold increased risk of acute coronary syndrome (ACS) and a 2.3-fold risk of cerebrovascular accident (CVA) among general patients (not encompassing patients with cardiovascular disease).^48^ Our meta-analysis added that high NLR doubled the risk of adverse outcomes among patients with PAD, including ACM, MALE, and MACE (Figures 2 and 4). This increased risk was notably more pronounced for in-hospital and 1-year analysis across those outcomes (Figures 2 and 4). In comparison, high NLR is associated with a 4.8-fold increase in mortality and 3.7-fold INCREASE in MACE among patients with ACS.^49^ Several plausible biological explanations might be offered in order to understand the above-mentioned findings. High NLR is associated with vulnerable atherosclerotic plaque.^50,51^ In aortic disease, high NLR is associated with higher short-term mortality compared to the low NLR group,^52^ which was also observed in patients who underwent surgical correction.^53^ Our meta-analysis also indirectly showed that high NLR is associated with worse outcomes among patients with PAD regardless of whether the intervention was performed or not (Table 1). This emphasizes the prognostic significance of high NLR across various atherosclerotic conditions, including PAD.

Furthermore, this present review is concerned with the association between high NLR and ACM that decreased as the follow-up extended. For instance, the risk of 1-year mortality was 1.6-fold (RR 2.5 vs 1.5) higher than 5-year ACM (Figure 2). A similar pattern was also observed in MALE outcomes (Figure 4), in which the risk of 1-year MALE was threefold (RR 3.9 vs 1.29) higher than 4-year MALE. Indeed, atherosclerosis involves chronic inflammation. However, the degree of inflammation varied across distinct conditions.^54^ Therefore, higher intensity of inflammation reflected by high NLR is more associated with short-term compared to long-term outcomes.

In addition to the association between high NLR and outcomes in patients with PAD, we also evaluated the predictive performance of NLR to predict ACM and MALE among patients with PAD. The performance of high NLR to predict ACM is moderate-to-good depending on the timeframe of follow-up. Though the overall performance of high NLR was found to be 36–58% in sensitivity and 73–84% specificity, the optimum 58% sensitivity and 73% specificity, as well as 0.7 AUC, were achieved when NLR was used to predict 1-year ACM (Figure S2, Figure S4, and Table 3). The performance of high NLR to predict ACM decreased as the duration of follow-up increased; however, the specificity remained constant at 70–83% (Table 3). Additionally, with the pooled prevalence of ACM at 41% and 17.33% in high and low NLR groups, the PPV and NPV of this parameter were 41% and 83%, respectively, in predicting 1-year ACM (Table 3). The performance of high NLR to predict MALE has a similar pattern when it is used to predict ACM; however, it remained better at AUC (0.74–0.78) irrespective of the follow-up duration (Table 3). The best performance of high NLR to predict MALE is observed at 1-year follow-up, with 65% sensitivity, 78% specificity, 54% PPV, 84% NPV, and 0.78 AUC (Table 3). Similarly, though meta-analysis could not be performed, high NLR performed better with higher sensitivity to predict in-hospital MACE (72%), and was more specific in predicting 1-year MACE (75%) (Figure S9). Therefore, high NLR is more sensitive in predicting short-term outcomes (than long-term outcomes) and more specific in predicting long-term outcomes (than short-term outcomes) in patients with PAD.

Nevertheless, it is worth noting that there was variation in the population characteristics of the included studies regarding the management of patients with PAD (Table 1). Patients undergoing revascularization surgery were expected to have different outcomes compared to those receiving nonoperative (medical) management.^55^ A cohort study engaging 15,314 patients with PAD in Germany (mean follow-up duration: 647 days) showed that limb amputation and overall mortality among patients with and without revascularization surgery were not quite different (limb amputation: 40.6% vs 46.5%; overall mortality: 42.6% vs 48.2%).^55^ Another study found that approximately 10% of patients undergoing endovascular revascularization required amputation after 180 days of follow-up, and 14.1% died within a year of revascularization.^56^ Meanwhile, a recent meta-analysis suggests that nonoperative (conservative) management can be considered and does not always result in more limb loss or patient demise.^57^ The pooled 12-month ACM rate for nonoperative management was 18%, and the pooled 12-month amputation rate was 27%.^57^

In our review, although most studies included patients undergoing revascularization surgery, a study by Luo et al.^58^ focused solely on patients with PAD managed nonoperatively (Table 1). Additionally, studies by Spark et al. and Erturk et al. included both patients with operative and nonoperative management, with Spark reporting 33% nonoperative cases and Erturk reporting 52% nonoperative cases.^39,43^ We assessed the influence of these studies in our pooled analysis using LOOS analysis. For the ACM outcome, omitting either Spark et al. or Erturk et al. did not significantly change the pooled outcome, except for the pooled risk ratio (Figure S1). Omitting Erturk et al. (with 52% receiving nonoperative management) reduced the heterogeneity (79% vs 32%, RR 2.05 vs RR 1.6) (Figure S1C). For the MALE outcome, Luo et al.^58^ only contributed in pooled 3-year analysis. Omitting Luo et al. did not result in a significant change in pooled heterogeneity and estimate, except for the pooled specificity in 3-year MALE (Figure S14). Omitting Luo et al. reduced the heterogeneity (83% vs 30%, specificity 81% vs 86%) (Figure S14C). In general, investigating the influence of patients’ management, in our case, showed a marked change of pooled outcomes.

To the best of our knowledge, this is the first comprehensive systematic review and meta-analysis evaluating the correlation between NLR and mortality, MACE, and MALE outcomes, alongside its performance in predicting these outcomes among patients with PAD. The early identification of high-risk patients with PAD holds paramount importance in enhancing outcomes through timely aggressive management.^22^ Our meta-analysis revealed that NLR could provide decent predictions for ACM and MALE occurrence among patients with PAD, particularly for short-term outcomes. Thus, NLR emerges as a cost-effective, feasible, and widely applicable prognostic biomarker for identifying high-risk patients with PAD.

### Limitations

We acknowledged several limitations to this study. Firstly, significant heterogeneity was observed across some follow-up durations. Attempts to address this included conducting subgroup and leave-out sensitivity analysis to try elucidating the observed heterogeneities. Secondly, the variability in the provided cut-offs for high and low NLR based on optimal AUC might have contributed to significant heterogeneity. Nonetheless, subgroup analysis indicated no substantial differences among different cut-off ranges. Although generally cut-offs between three and four demonstrated a tendency toward higher performance (albeit not statistically significant), we refrained from recommending specific cut-offs for clinical practice due to potential variations in optimal cut-offs across diverse populations or settings. Thirdly, the included population comprised patients with PAD with varying severity, predominantly falling into the moderate–severe category based on conventional assessments. However, limited data prevented subgroup analysis and publication bias analysis, posing concerns about the applicability of NLR’s predictive ability among mild patients with PAD. Therefore, further validation through individual participant data meta-analysis and robust large cohorts is important to confirm these findings.

## Conclusion

High NLR is associated with a higher risk of ACM, MALE, and MACE among patients with PAD. High NLR possesses good performance to predict ACM and MALE. The performance of NLR is best for predicting short-term outcomes and tends to decrease as the follow-up duration increases. The NLR’s sensitivity is decreased and the specificity is increased as the follow-up duration increases. In conclusion, NLR is a potential prognostic biomarker for identification of high-risk patients with PAD.

## Supplemental Material

sj-pdf-1-vmj-10.1177_1358863X241281699 – Supplemental material for Neutrophil-to-lymphocyte ratio as a prognostic biomarker in patients with peripheral artery disease: A systematic review and meta-analysis Supplemental material, sj-pdf-1-vmj-10.1177_1358863X241281699 for Neutrophil-to-lymphocyte ratio as a prognostic biomarker in patients with peripheral artery disease: A systematic review and meta-analysis by Roy B Kurniawan, Paulus P Siahaan, Pandit BT Saputra, Jannatin N Arnindita, Cornelia G Savitri, Novia N Faizah, Luqman H Andira, Mario D’Oria, J Nugroho Eko Putranto and Firas F Alkaff in Vascular Medicine
