# Supplementary material for: Neutrophil-to-lymphocyte ratio as a prognostic biomarker in patients with peripheral artery disease: A systematic review and meta-analysis
Source: Vasc Med. 2024 Oct 16;29(6):687–99. doi: 10.1177/1358863X241281699 (PMC11590382; doi:10.1177/1358863X241281699)

Supplementary Tables

Table S1 PRISMA checklist

Table S2 Literature search

Table S3 Quality assessment of included studies (Newcastle Ottawa Scale)

Supplementary Figures

Figure S1. Additional analyses for pooled risk in ACM outcome

A. Subgroup analysis of pooled risk in ACM outcome (1-year follow-up)

B. Baujat plot of pooled risk in ACM outcome (1-year follow-up)

C. Leave-one-out sensitivity analysis of pooled risk in ACM outcome. Top: 1-year follow-up, Bottom: 2-year follow-up.

Figure S2. Pooled sensitivity of high NLR to Predict ACM

Figure S3. Additional analyses for sensitivity in ACM outcome

A. Subgroup analysis of pooled sensititvity in ACM outcome (1-year follow-up)

B. Baujat plot of poolod sensitivity in ACM outcome (1-year follow-up)

C. Leave-one-out sensitivity analysis of pooled sensitivity in the ACM outcome. Top: 1-year follow-up, Bottom: 2-year follow-up.

Figure S4. Pooled specificity of high NLR to Predict ACM

Figure S5. Additional analyses for specificity in ACM outcome

A. Subgroup analysis of pooled specificity in ACM outcome (1-year follow-up)

B. Baujat plot of poolod specificity in ACM outcome (1-year follow-up)

C. Leave-one-out sensitivity analysis of pooled specificity in the ACM outcome. Top: 1-year follow-up, Bottom: 2-year follow-up.

Figure S6. Pooled prevalence of ACM between high and low NLR group

Figure S7. Pooled PPV of high NLR to predict ACM

Figure S8. Pooled NPV of high NLR to predict ACM

Figure S9. NLR and MACE association (TOP) and its sensitivity and specificity top Predict MACE (Bottom)

Figure S10. Additional analyses for pooled risk in MALE outcome

A. Subgroup analysis of pooled risk in MALE Outcome (1-year follow-up)

B. Baujat plot of pooled risk in MALE Outcome (1-year follow-up)

C. Leave-one-out sensitivity analysis of pooled risk in MALE outcome. Top: 1-year follow-up, Bottom: 3-year follow-up.

Figure S11. Pooled sensitivity of high NLR to Predict MALE

Figure S12. Additional analyses for sensitivity in MALE outcome

A. Subgroup Analysis of Pooled Sensititvity in MALE outcome (1-year follow-up)

B. Baujat Plot of Poolod Sensitivity in MALE outcome (1-year follow-up)

C. Leave-one-out sensitivity analysis of Pooled Sensitivity in the MALE outcome. Top: 1-year follow-up, Bottom: 3-year follow-up.

Figure S13. Pooled specificity of high NLR to Predict MALE

Figure S14. Additional analyses for specificity in MALE outcome

A. Subgroup analysis of pooled specificity in MALE outcome (1-year follow-up)

B. Leave-one-out sensitivity analysis of pooled specificity in the MALE outcome. Top: 1-year follow-up, Bottom: 3-year follow-up.

Figure S15. Pooled prevalence of MALE between high and low NLR group

Figure S16. Pooled PPV of high NLR to predict MALE

Figure S17. Pooled NPV of high NLR to predict MALE

Table S1. PRISMA checklist

Section and Topic	Item #	Checklist item	Location where item is reported
**TITLE**			
Title	1	Identify the report as a systematic review.	p.1
**ABSTRACT**			
Abstract	2	See the PRISMA 2020 for Abstracts checklist.	p. 3
**INTRODUCTION**			
Rationale	3	Describe the rationale for the review in the context of existing knowledge.	p. 3
Objectives	4	Provide an explicit statement of the objective(s) or question(s) the review addresses.	p. 4
**METHODS**			
Eligibility criteria	5	Specify the inclusion and exclusion criteria for the review and how studies were grouped for the syntheses.	p.5
Information sources	6	Specify all databases, registers, websites, organisations, reference lists and other sources searched or consulted to identify studies. Specify the date when each source was last searched or consulted.	p.5
Search strategy	7	Present the full search strategies for all databases, registers and websites, including any filters and limits used.	p.5
Selection process	8	Specify the methods used to decide whether a study met the inclusion criteria of the review, including how many reviewers screened each record and each report retrieved, whether they worked independently, and if applicable, details of automation tools used in the process.	p. 5-6
Data collection process	9	Specify the methods used to collect data from reports, including how many reviewers collected data from each report, whether they worked independently, any processes for obtaining or confirming data from study investigators, and if applicable, details of automation tools used in the process.	p.6
Data items	10a	List and define all outcomes for which data were sought. Specify whether all results that were compatible with each outcome domain in each study were sought (e.g. for all measures, time points, analyses), and if not, the methods used to decide which results to collect.	p.6
	10b	List and define all other variables for which data were sought (e.g. participant and intervention characteristics, funding sources). Describe any assumptions made about any missing or unclear information.	p.6
Study risk of bias assessment	11	Specify the methods used to assess risk of bias in the included studies, including details of the tool(s) used, how many reviewers assessed each study and whether they worked independently, and if applicable, details of automation tools used in the process.	p.6
Effect measures	12	Specify for each outcome the effect measure(s) (e.g. risk ratio, mean difference) used in the synthesis or presentation of results.	p.6-7
Synthesis methods	13a	Describe the processes used to decide which studies were eligible for each synthesis (e.g. tabulating the study intervention characteristics and comparing against the planned groups for each synthesis (item #5)).	p.7

Section and Topic	Item #	Checklist item	Location where item is reported
	13b	Describe any methods required to prepare the data for presentation or synthesis, such as handling of missing summary statistics, or data conversions.	p.7
	13c	Describe any methods used to tabulate or visually display results of individual studies and syntheses.	p.7
	13d	Describe any methods used to synthesize results and provide a rationale for the choice(s). If meta-analysis was performed, describe the model(s), method(s) to identify the presence and extent of statistical heterogeneity, and software package(s) used.	p.7
	13e	Describe any methods used to explore possible causes of heterogeneity among study results (e.g. subgroup analysis, meta-regression).	p.7
	13f	Describe any sensitivity analyses conducted to assess robustness of the synthesized results.	p.7
Reporting bias assessment	14	Describe any methods used to assess risk of bias due to missing results in a synthesis (arising from reporting biases).	p.7
Certainty assessment	15	Describe any methods used to assess certainty (or confidence) in the body of evidence for an outcome.	N/A
**RESULTS**			
Study selection	16a	Describe the results of the search and selection process, from the number of records identified in the search to the number of studies included in the review, ideally using a flow diagram.	p.8
	16b	Cite studies that might appear to meet the inclusion criteria, but which were excluded, and explain why they were excluded.	p.8
Study characteristics	17	Cite each included study and present its characteristics.	p.8
Risk of bias in studies	18	Present assessments of risk of bias for each included study.	p.8
Results of individual studies	19	For all outcomes, present, for each study: (a) summary statistics for each group (where appropriate) and (b) an effect estimate and its precision (e.g. confidence/credible interval), ideally using structured tables or plots.	p. 11-17
Results of syntheses	20a	For each synthesis, briefly summarise the characteristics and risk of bias among contributing studies.	p. 17
	20b	Present results of all statistical syntheses conducted. If meta-analysis was done, present for each the summary estimate and its precision (e.g. confidence/credible interval) and measures of statistical heterogeneity. If comparing groups, describe the direction of the effect.	p. 11-18
	20c	Present results of all investigations of possible causes of heterogeneity among study results.	p. 11-19
	20d	Present results of all sensitivity analyses conducted to assess the robustness of the synthesized results.	p. 11-19
Reporting biases	21	Present assessments of risk of bias due to missing results (arising from reporting biases) for each synthesis assessed.	N/A

Section and Topic	Item #	Checklist item	Location where item is reported
Certainty of evidence	22	Present assessments of certainty (or confidence) in the body of evidence for each outcome assessed.	N/A
**DISCUSSION**			
Discussion	23a	Provide a general interpretation of the results in the context of other evidence.	p. 18-20
	23b	Discuss any limitations of the evidence included in the review.	p. 20
	23c	Discuss any limitations of the review processes used.	p. 20
	23d	Discuss implications of the results for practice, policy, and future research.	p. 20
**OTHER INFORMATION**			
Registration and protocol	24a	Provide registration information for the review, including register name and registration number, or state that the review was not registered.	p. 5
	24b	Indicate where the review protocol can be accessed, or state that a protocol was not prepared.	p. 5
	24c	Describe and explain any amendments to information provided at registration or in the protocol.	N/A
Support	25	Describe sources of financial or non-financial support for the review, and the role of the funders or sponsors in the review.	p. 21
Competing interests	26	Declare any competing interests of review authors.	p. 21
Availability of data, code and other materials	27	Report which of the following are publicly available and where they can be found: template data collection forms; data extracted from included studies; data used for all analyses; analytic code; any other materials used in the review.	N/A

Table S2. Literature search

#1 (("PAD") OR ("peripheral artery disease") OR ("peripheral arterial disease") OR ("intermittent claudication") OR ("limb ischemia"))

#2 (("Neutrophil to Lymphocyte") OR ("Neutrophil to Lymphocyte Ratio") OR ("Neutrophil-to-Lymphocyte Ratio") OR ("NLR"))

**Database**	**Keywords**	**Search Results**	**Search Time**
PubMed	#1 AND #2	102	January 1, 2024 (20:20, GMT+7)
Science Direct	#1 AND #2	29	January 1, 2024 (20:20, GMT+7)
Scopus	#1 AND #2	1414	January 1, 2024 (20:20, GMT+7)
Web of Science	#1 AND #2	102	January 1, 2024 (20:20, GMT+7)
ProQuest	#1 AND #2	1468	January 1, 2024 (20:20, GMT+7)
EBSCO	#1 AND #2	20	January 1, 2024 (20:20, GMT+7)
Cochrane	#1 AND #2	2	January 1, 2024 (20:20, GMT+7)

Table S3. Quality Assessment of Included Studies (Newcastle Ottawa Scale)

Study	Ref.	Selection				Comparability	Outcome			Total (8/8)	Conclusion
		A	B	C	D	E	F	G	H		
**Cohort studies**											
Adler, 2022	[42]	o	★	★	★	o	★	★	o	5	Moderate
Bath, 2019	[40]	★	★	★	★	o	★	★	o	6	Moderate
Ertruk, 2014	[43]	o	★	★	★	★	★	★	o	6	Moderate
Gonzalez-Fajardo, 2014	[45]	★	★	★	★	★	★	★	o	7	High
King, 2021	[41]	★	★	★	★	o	★	★	o	6	Moderate
Luo, 2015	[58]	o	★	★	★	★	★	★	o	6	Moderate
Russu, 2022	[44]	o	★	★	★	★	★	★	★	7	High
Spark, 2010	[39]	o	★	★	★	★	★	★	★	7	High
Su, 2021	[46]	o	★	★	★	o	★	★	o	5	Moderate

Figure S1. Additional analyses for pooled risk in ACM outcome

A. Subgroup analysis of pooled risk in ACM outcome (1-year follow-up)

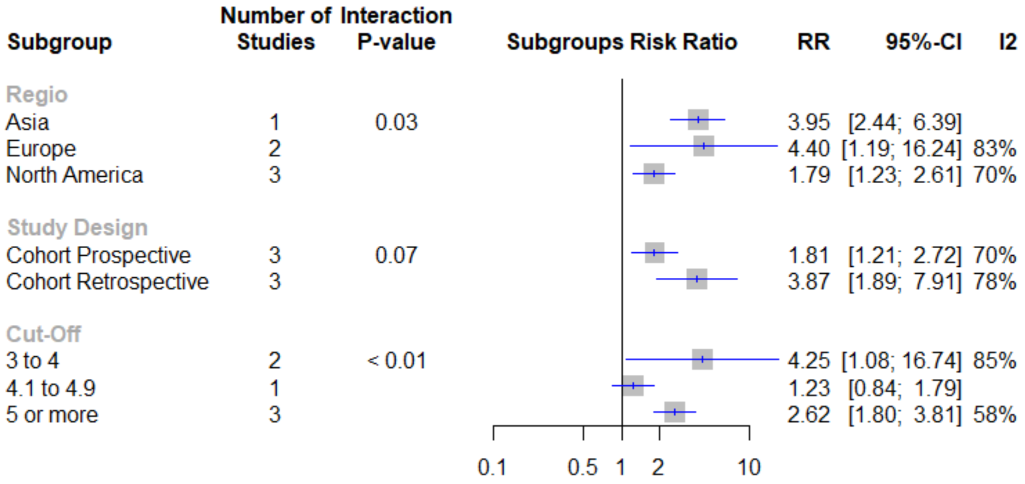

B. Baujat plot of pooled risk in ACM outcome (1-year follow-up)

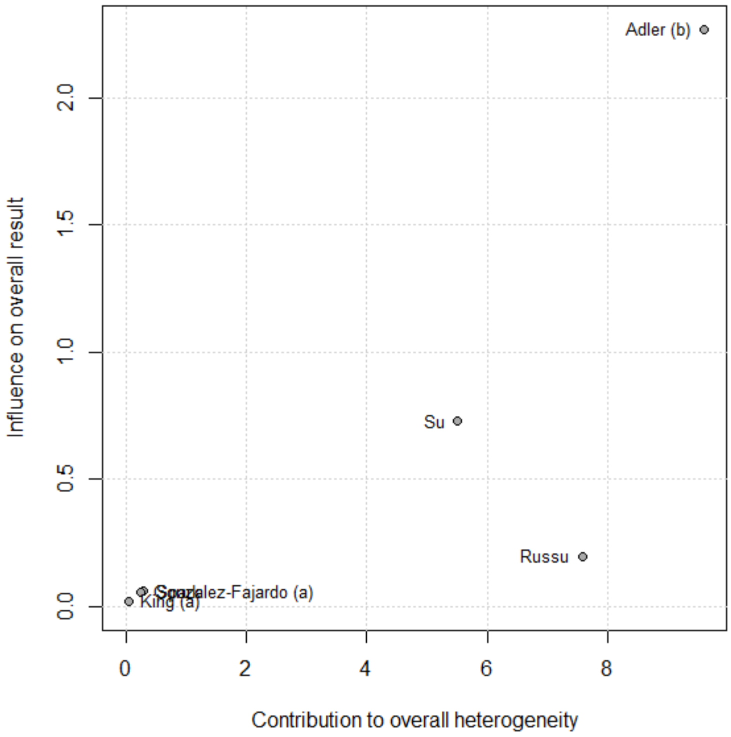

Adler (b)
Su
Russu
Gonzalez-Fajardo (a)
Influence on overall result
Contribution to overall heterogeneity
0.0
0.5
1.0
1.5
2.0
0
2
4
6
8

C. Leave-one-out sensitivity analysis of pooled risk in ACM outcome. Top: 1-year follow-up, Bottom: 2-year follow-up.

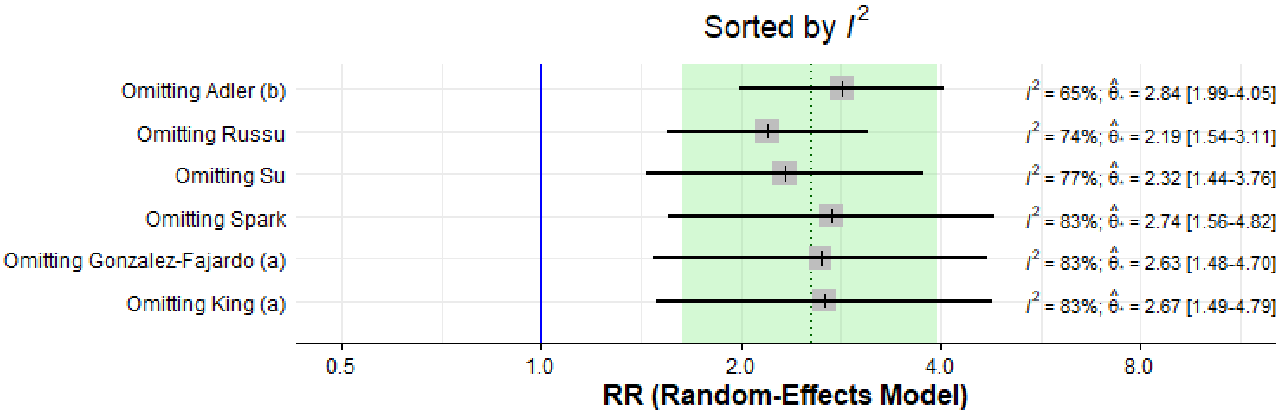

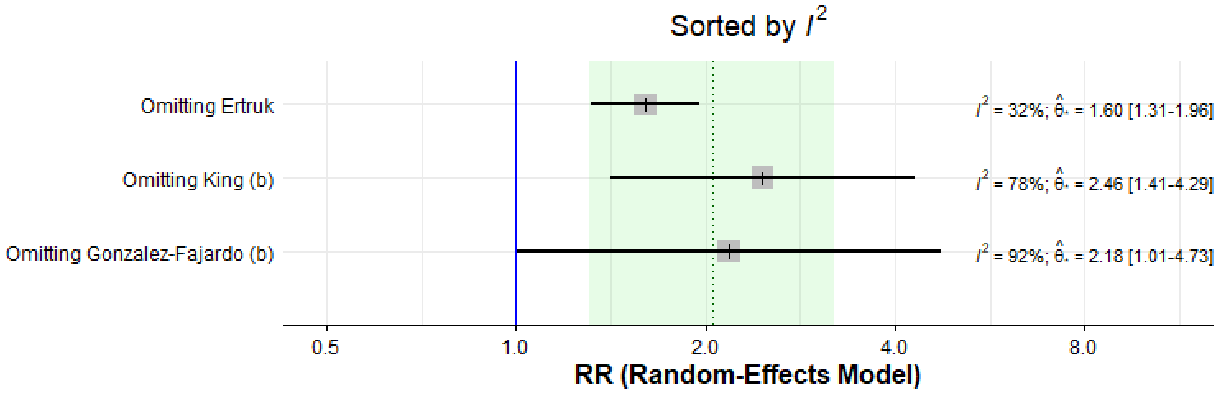

Figure S2. Pooled sensitivity of high NLR to predict ACM

Study	Ref.	Events	Total	Sensitivity (%)	95% CI
**Inhospital ACM**					
Bath	[40]	17	20	85.00	[62.11; 96.79]
**30−day ACM**					
Adler (a)	[42]	21	55	38.18	[25.41; 52.27]
**1−year ACM**					
Adler (b)	[42]	32	70	45.71	[33.74; 58.06]
Spark	[39]	38	62	61.29	[48.07; 73.40]
Gonzalez−Fajardo (a)	[45]	33	87	37.93	[27.74; 48.97]
King (a)	[41]	77	131	58.78	[49.85; 67.30]
Su	[46]	31	50	62.00	[47.17; 75.35]
Russu	[44]	23	27	85.19	[66.27; 95.81]
**Random effects model**			**427**	**58.15**	**[45.34; 70.97]**
Heterogeneity: I^2 = 86%, τ^2 = 0.0221, p < 0.01					
**2−year ACM**					
Gonzalez−Fajardo (b)	[45]	38	119	31.93	[23.69; 41.10]
King (b)	[41]	127	263	48.29	[42.11; 54.51]
Ertruk	[43]	43	75	57.33	[45.38; 68.69]
**Random effects model**			**457**	**45.58**	**[31.35; 59.80]**
Heterogeneity: I^2 = 87%, τ^2 = 0.0139, p < 0.01					
**3−year ACM**					
Gonzalez−Fajardo (c)	[45]	47	146	32.19	[24.71; 40.42]
King (c)	[41]	155	348	44.54	[39.24; 49.93]
**Random effects model**			**494**	**38.68**	**[26.60; 50.77]**
Heterogeneity: I^2 = 86%, τ^2 = 0.0065, p < 0.01					
**4−year ACM**					
Gonzalez−Fajardo (d)	[45]	51	182	28.02	[21.63; 35.14]
King (d)	[41]	172	421	40.86	[36.12; 45.72]
**Random effects model**			**603**	**34.65**	**[22.08; 47.22]**
Heterogeneity: I^2 = 90%, τ^2 = 0.0074, p < 0.01					
**5−year ACM**					
Adler (c)	[42]	39	84	46.43	[35.47; 57.65]
Gonzalez−Fajardo (e)	[45]	55	203	27.09	[21.11; 33.76]
**Random effects model**			**287**	**36.25**	**[17.33; 55.17]**
Heterogeneity: I^2 = 89%, τ^2 = 0.0167, p < 0.01					

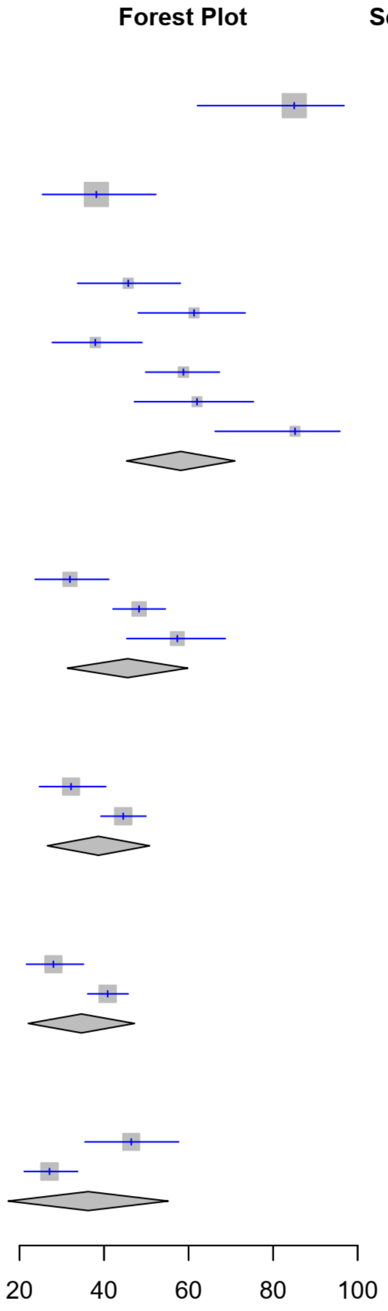

Figure S3. Additional analyses for sensitivity in ACM outcome

A. Subgroup analysis of pooled sensitivity in ACM outcome (1-year follow-up)

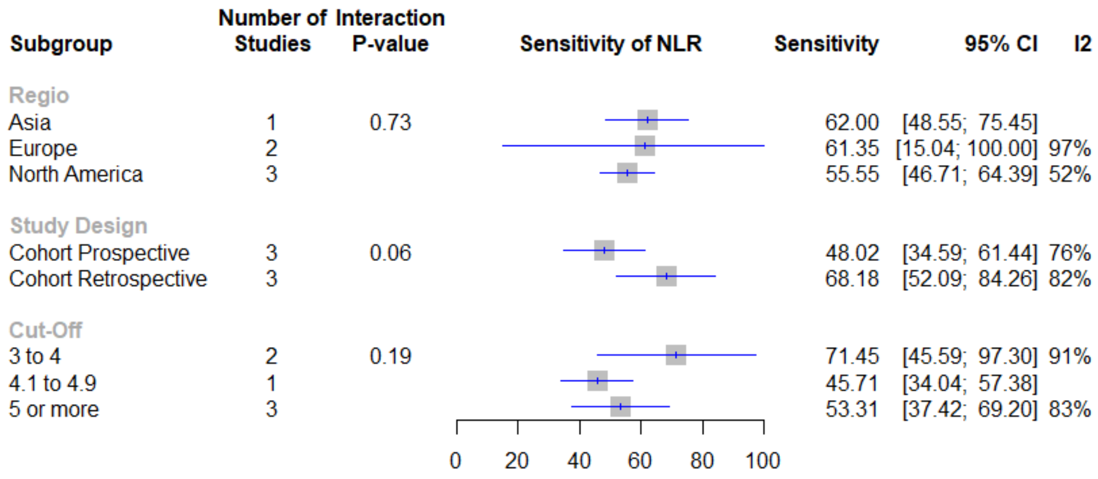

B. Baujat plot of poolod sensitivity in ACM outcome (1-year follow-up)

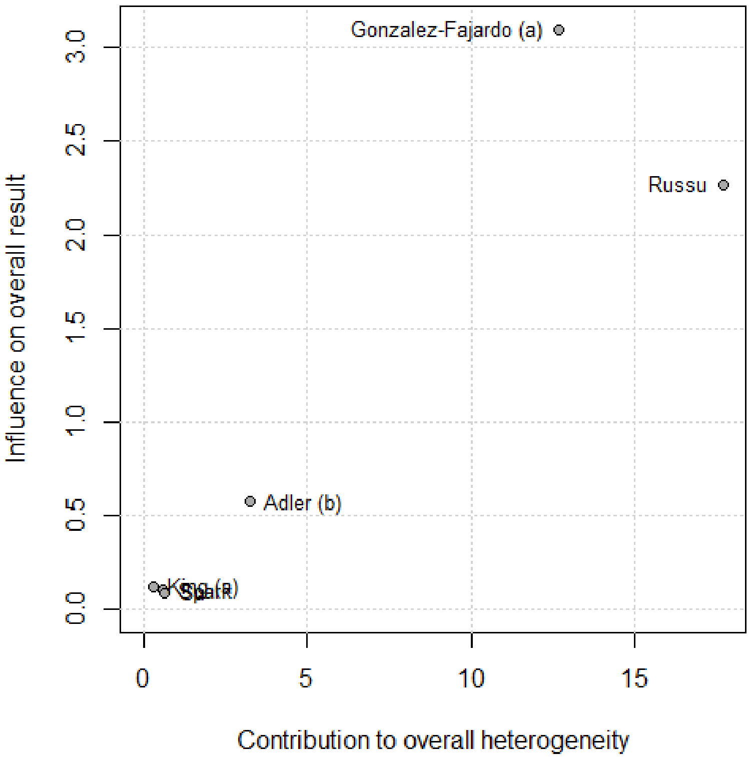

Influence on overall result
Contribution to overall heterogeneity
Gonzalez-Fajardo (a)
Russu
Adler (b)
0.0
0.5
1.0
1.5
2.0
2.5
3.0
0
5
10
15

C. Leave-one-out sensitivity analysis of pooled sensitivity in the ACM outcome. Top: 1-year follow-up, Bottom: 2-year follow-up.

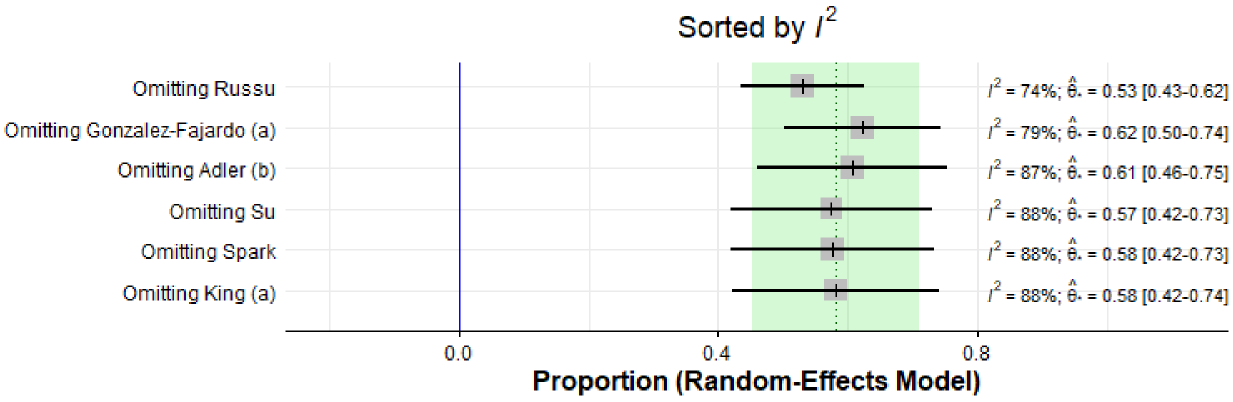

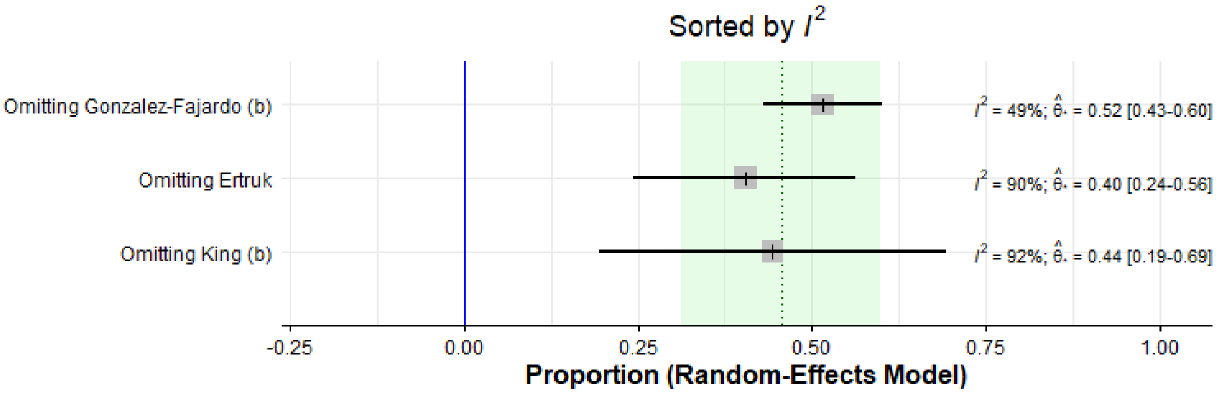

Figure S4. Pooled specificity of high NLR to predict ACM

Study	Ref.	Events	Total	Specificity (%)	95% CI
**Inhospital ACM**					
Bath	[40]	936	2681	34.91	[33.11; 36.75]
**30−day ACM**					
Adler (a)	[42]	118	199	59.30	[52.12; 66.19]
**1−year ACM**					
Adler (b)	[42]	80	129	62.02	[53.05; 70.41]
Spark	[39]	59	87	67.82	[56.94; 77.44]
Gonzalez−Fajardo (a)	[45]	395	474	83.33	[79.67; 86.58]
King (a)	[41]	247	357	69.19	[64.11; 73.94]
Su	[46]	119	145	82.07	[74.84; 87.94]
Russu	[44]	135	197	68.53	[61.55; 74.94]
**Random effects model**			**1389**	**72.63**	**[65.64; 79.62]**
Heterogeneity: $I^2 = 90\%$, $\tau^2 = 0.0065$, $p < 0.01$					
**2−year ACM**					
Gonzalez−Fajardo (b)	[45]	368	442	83.26	[79.44; 86.62]
King (b)	[41]	165	225	73.33	[67.05; 78.99]
Ertruk	[43]	294	383	76.76	[72.20; 80.90]
**Random effects model**			**1050**	**78.11**	**[72.38; 83.85]**
Heterogeneity: $I^2 = 81\%$, $\tau^2 = 0.0020$, $p < 0.01$					
**3−year ACM**					
Gonzalez−Fajardo (c)	[45]	350	415	84.34	[80.48; 87.70]
King (c)	[41]	108	140	77.14	[69.29; 83.81]
**Random effects model**			**555**	**81.39**	**[74.46; 88.33]**
Heterogeneity: $I^2 = 70\%$, $\tau^2 = 0.0018$, $p = 0.07$					
**4−year ACM**					
Gonzalez−Fajardo (d)	[45]	318	379	83.91	[79.81; 87.46]
King (d)	[41]	52	67	77.61	[65.78; 86.89]
**Random effects model**			**446**	**82.54**	**[77.45; 87.62]**
Heterogeneity: $I^2 = 26\%$, $\tau^2 = 0.0005$, $p = 0.25$					
**5−year ACM**					
Adler (c)	[42]	35	45	77.78	[62.91; 88.80]
Gonzalez−Fajardo (e)	[45]	301	358	84.08	[79.87; 87.71]
**Random effects model**			**403**	**83.52**	**[79.90; 87.14]**
Heterogeneity: $I^2 = 0\%$, $\tau^2 = 0$, $p = 0.33$					

Forest Plot

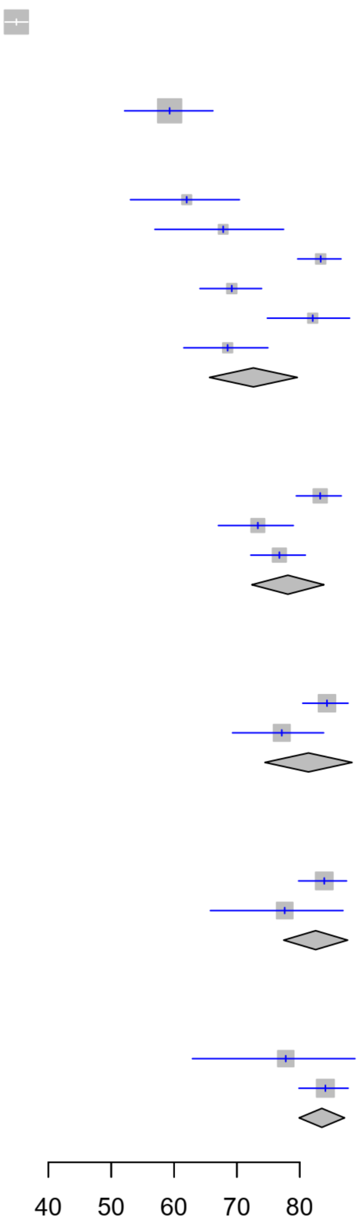

Figure S5. Additional analyses for specificity in ACM outcome

A. Subgroup analysis of pooled specificity in ACM outcome (1-year follow-up)

Subgroup	Number of Studies	Interaction P-value	Specificity of NLR	Specificity	95% CI	I2
**Regio**						
Asia	1	< 0.01		82.07	[75.83; 88.31]	
Europe	2			76.20	[61.70; 90.70]	94%
North America	3			67.23	[62.85; 71.60]	6%
**Study Design**						
Cohort Prospective	3	0.84		71.61	[58.56; 84.66]	93%
Cohort Retrospective	3			73.18	[64.64; 81.71]	84%
**Cut-Off**						
3 to 4	2	0.03		68.95	[65.10; 72.81]	0%
4.1 to 4.9	1			62.02	[53.64; 70.39]	
5 or more	3			78.68	[69.88; 87.49]	77%

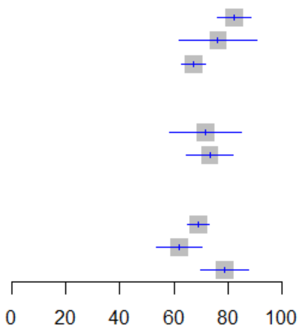

B. Baujat plot of poolod specificity in ACM outcome (1-year follow-up)

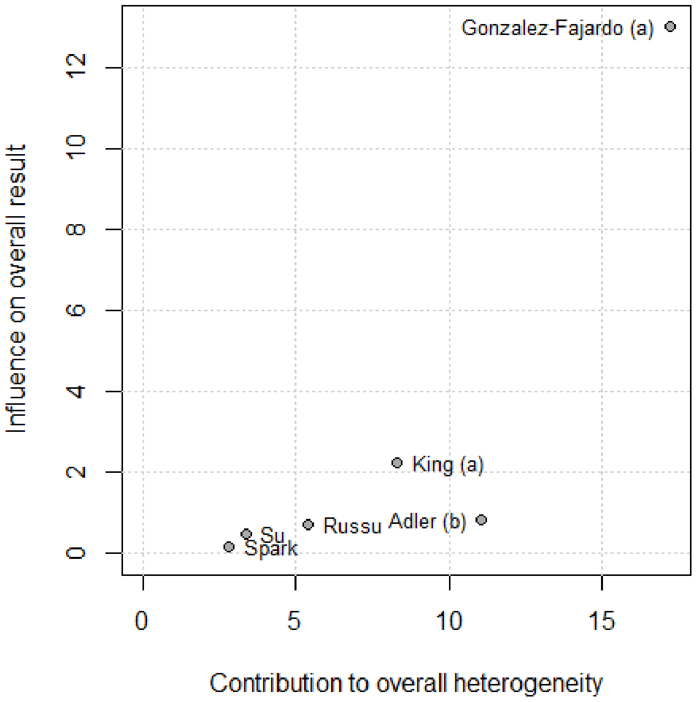

C. Leave-one-out sensitivity analysis of pooled specificity in the ACM outcome. Top: 1-year follow-up, Bottom: 2-year follow-up.

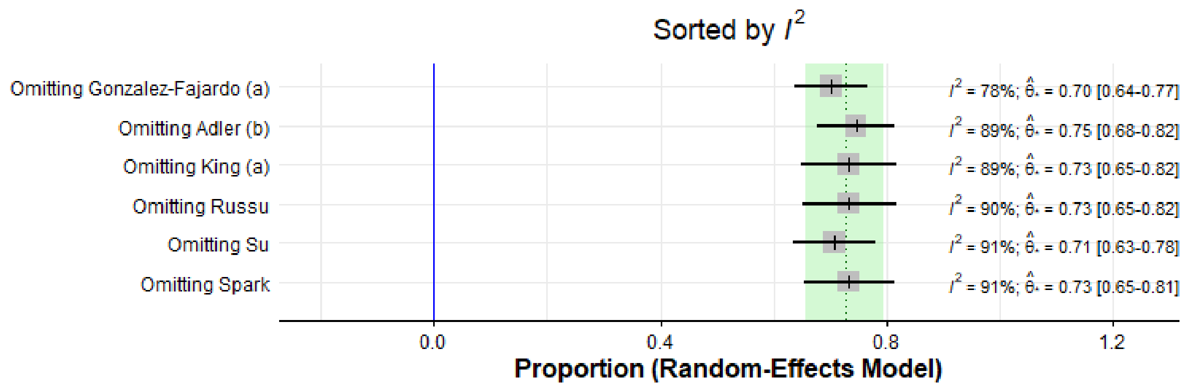

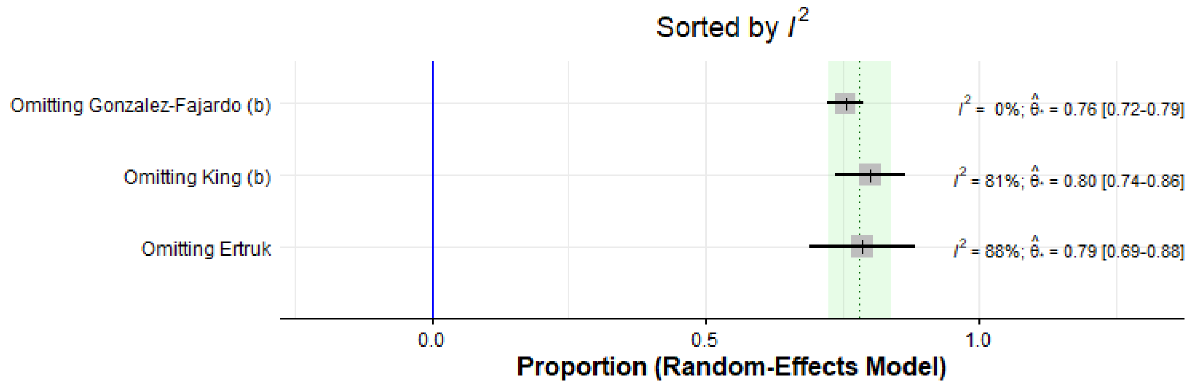

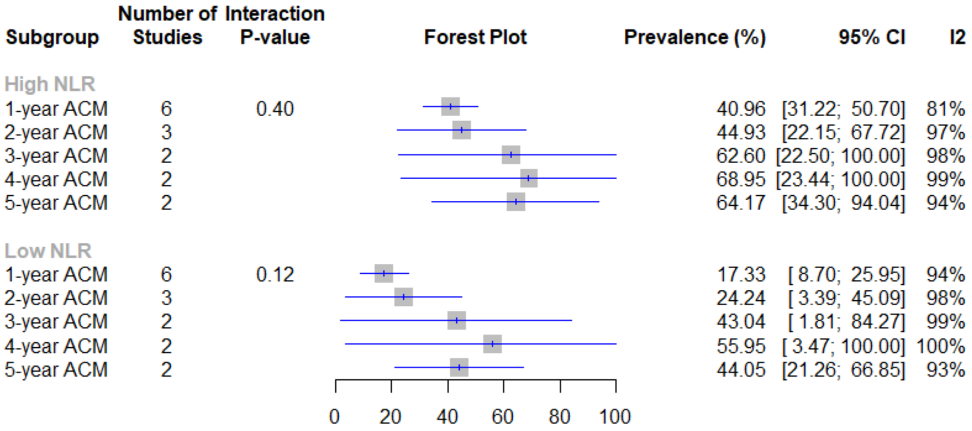

Figure S6. Pooled prevalence of ACM between high and low NLR group

Figure S7. Pooled PPV of high NLR to predict ACM

Study	Ref.	Events	Total	PPV (%)	95% CI
**Inhospital ACM**					
Bath	[40]	17	1762	0.96	[0.56; 1.54]
**30−day ACM**					
Adler (a)	[42]	21	102	20.59	[13.22; 29.73]
**1−year ACM**					
Adler (b)	[42]	32	81	39.51	[28.81; 50.99]
Spark	[39]	38	66	57.58	[44.79; 69.66]
Gonzalez−Fajardo (a)	[45]	33	112	29.46	[21.23; 38.82]
King (a)	[41]	77	187	41.18	[34.05; 48.59]
Su	[46]	31	57	54.39	[40.66; 67.64]
Russu	[44]	23	85	27.06	[17.99; 37.79]
**Random effects model**			588	40.96	[31.22; 50.70]
Heterogeneity: $I^2 = 81\%$, $\tau^2 = 0.0121$, $p < 0.01$					
**2−year ACM**					
Gonzalez−Fajardo (b)	[45]	38	112	33.93	[25.25; 43.48]
King (b)	[41]	127	187	67.91	[60.71; 74.54]
Ertruk	[43]	43	132	32.58	[24.68; 41.27]
**Random effects model**			431	44.93	[22.15; 67.72]
Heterogeneity: $I^2 = 97\%$, $\tau^2 = 0.0389$, $p < 0.01$					
**3−year ACM**					
Gonzalez−Fajardo (c)	[45]	47	112	41.96	[32.70; 51.66]
King (c)	[41]	155	187	82.89	[76.71; 87.99]
**Random effects model**			299	62.60	[22.50; 100.00]
Heterogeneity: $I^2 = 98\%$, $\tau^2 = 0.0823$, $p < 0.01$					
**4−year ACM**					
Gonzalez−Fajardo (d)	[45]	51	112	45.54	[36.10; 55.22]
King (d)	[41]	172	187	91.98	[87.12; 95.44]
**Random effects model**			299	68.95	[23.44; 100.00]
Heterogeneity: $I^2 = 99\%$, $\tau^2 = 0.1065$, $p < 0.01$					
**5−year ACM**					
Adler (c)	[42]	39	49	79.59	[65.66; 89.76]
Gonzalez−Fajardo (e)	[45]	55	112	49.11	[39.54; 58.73]
**Random effects model**			161	64.17	[34.30; 94.04]
Heterogeneity: $I^2 = 94\%$, $\tau^2 = 0.0437$, $p < 0.01$					

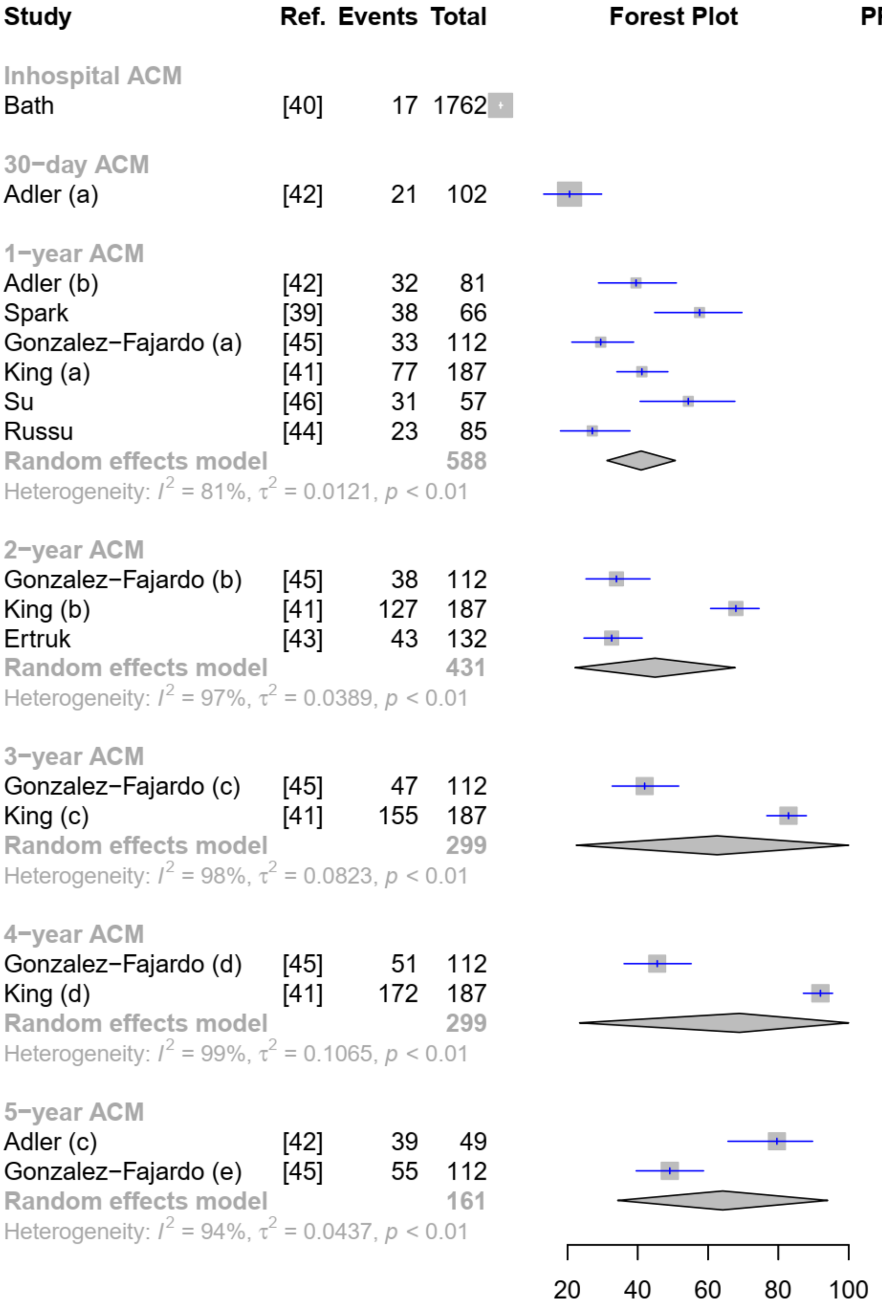

Figure S8. Pooled NPV of high NLR to predict ACM

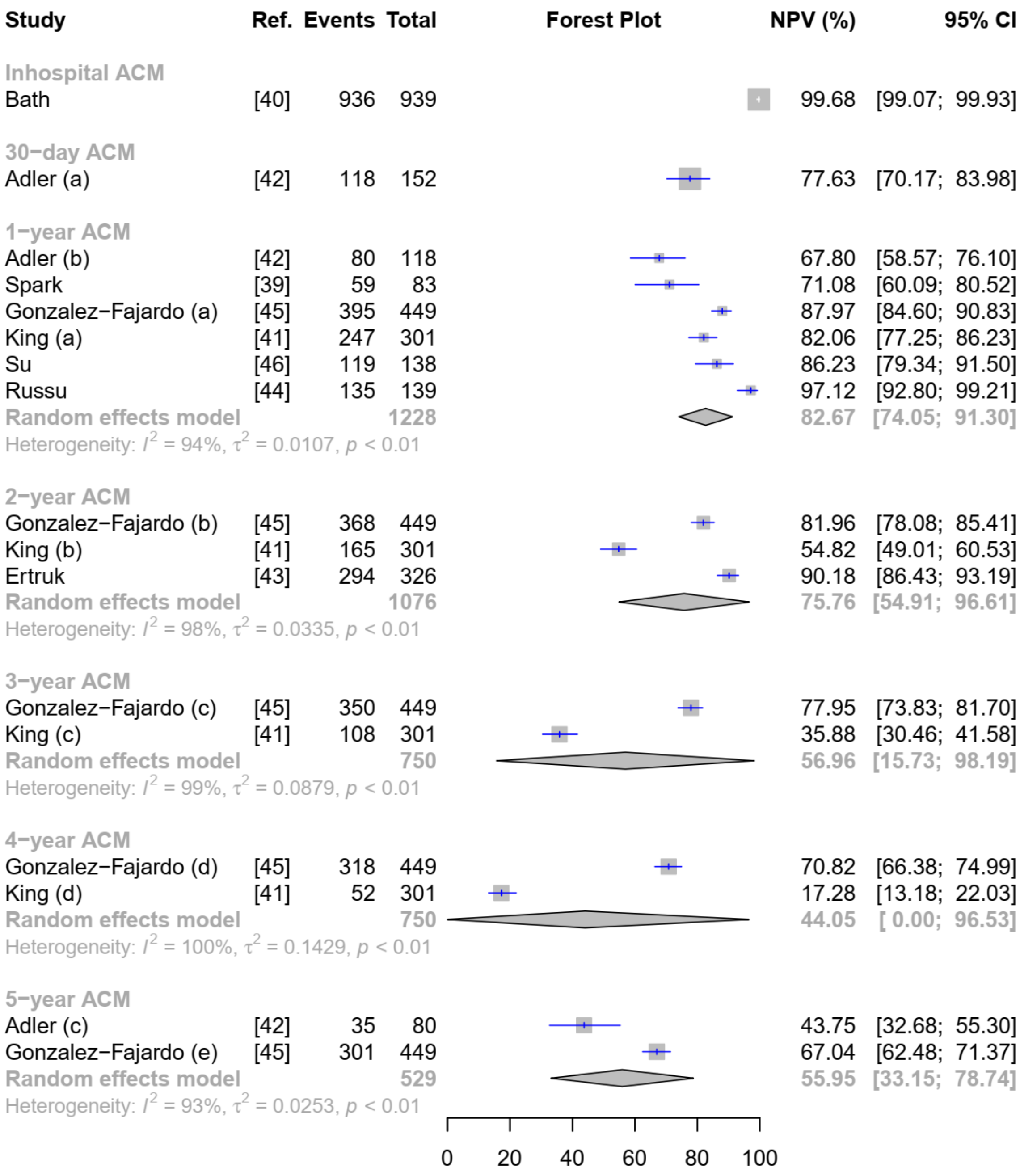

Figure S9. Forest plot of NLR and MACE association

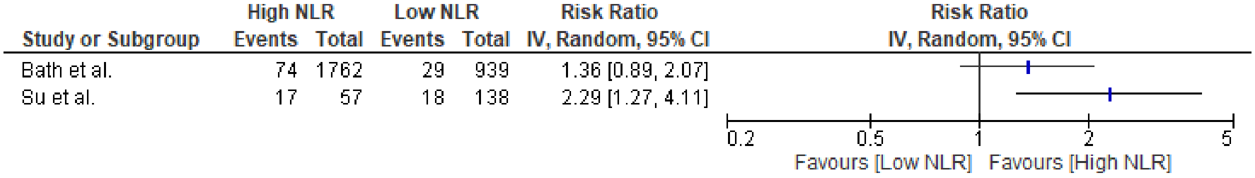

Figure S10. Additional analyses for pooled risk in MALE outcome

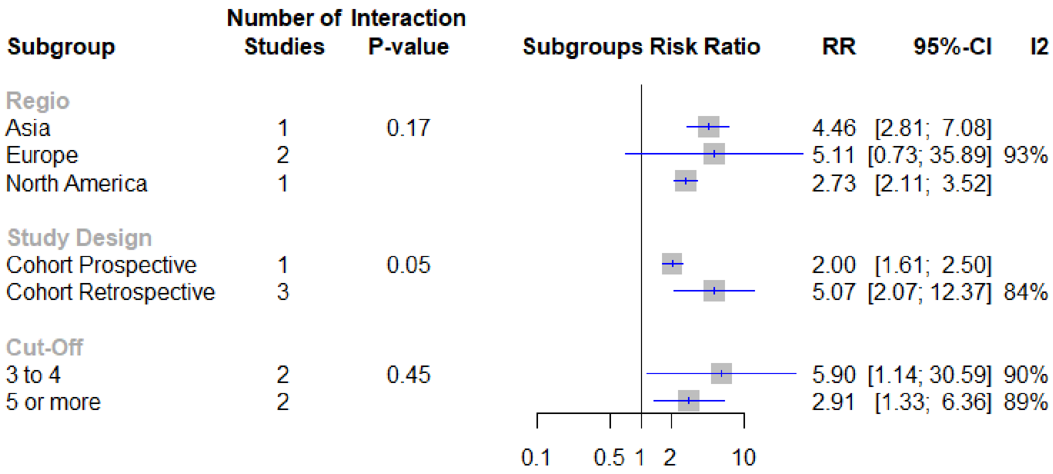

A. Subgroup analysis of pooled risk in MALE outcome (1-year follow-up)

B. Baujat plot of pooled risk in MALE outcome (1-year follow-up)

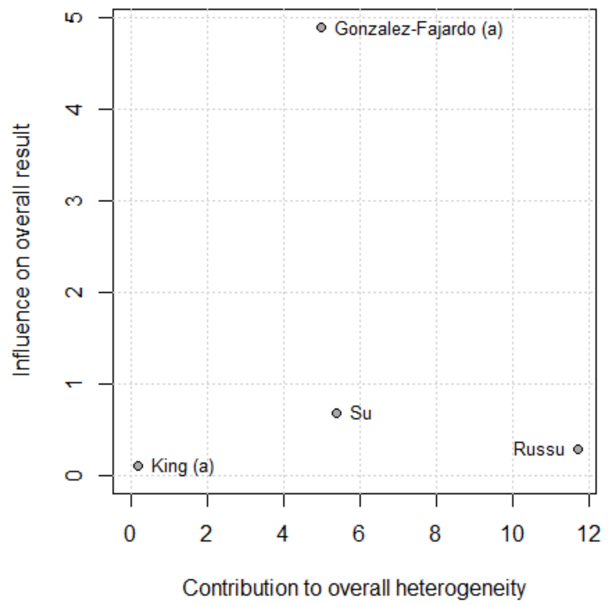

C. Leave-one-out sensitivity analysis of pooled risk in MALE outcome. Top: 1-year follow-up, Bottom: 3-year follow-up.

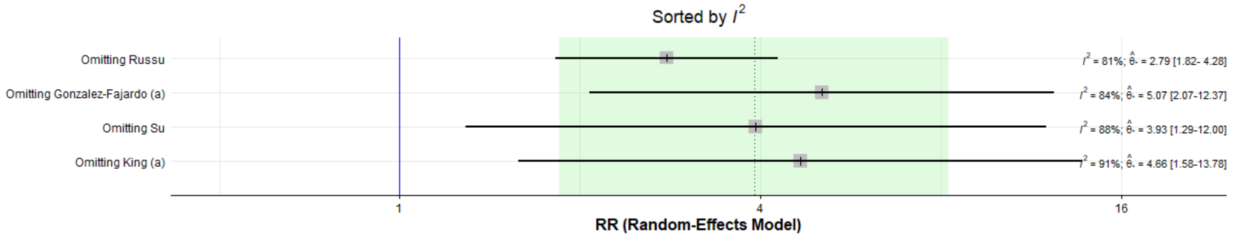

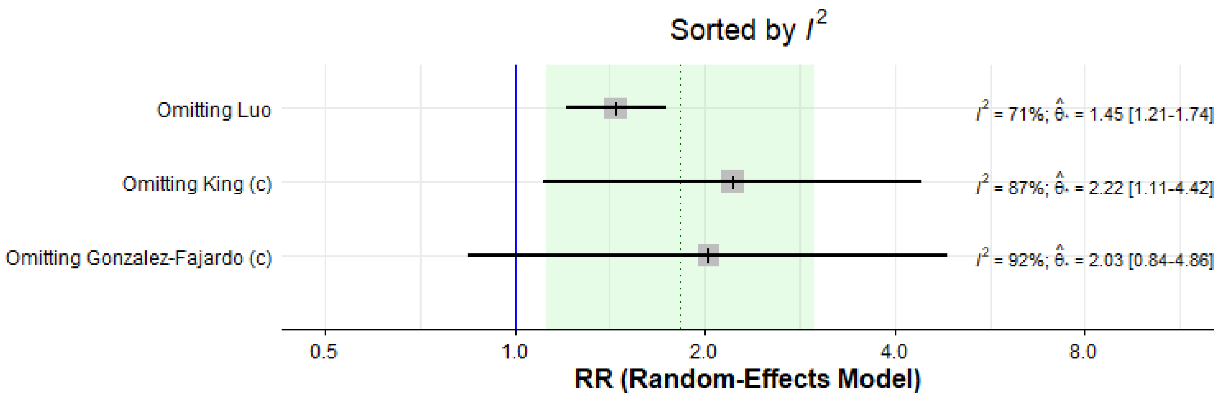

Sorted by I^2
Omitting Luo
Omitting King (c)
Omitting Gonzalez-Fajardo (c)
I^2 = 71%; $\hat{\theta}$ = 1.45 [1.21-1.74]
I^2 = 87%; $\hat{\theta}$ = 2.22 [1.11-4.42]
I^2 = 92%; $\hat{\theta}$ = 2.03 [0.84-4.86]
0.5
1.0
2.0
4.0
8.0
RR (Random-Effects Model)

Figure S11. Pooled sensitivity of high NLR to predict MALE

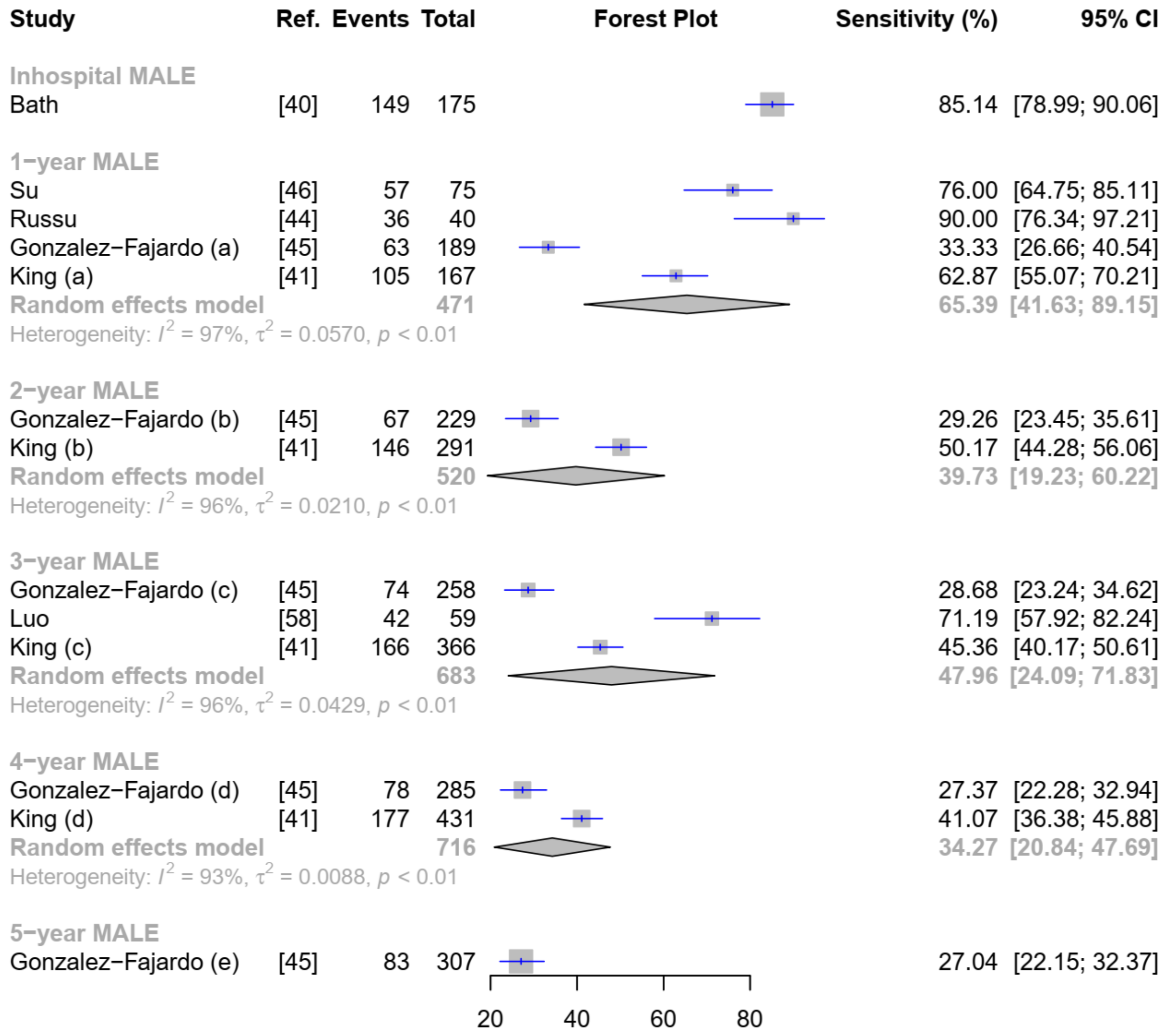

Figure S12. Additional analyses for sensitivity in MALE outcome

A. Subgroup analysis of pooled sensitivity in MALE outcome (1-year follow-up)

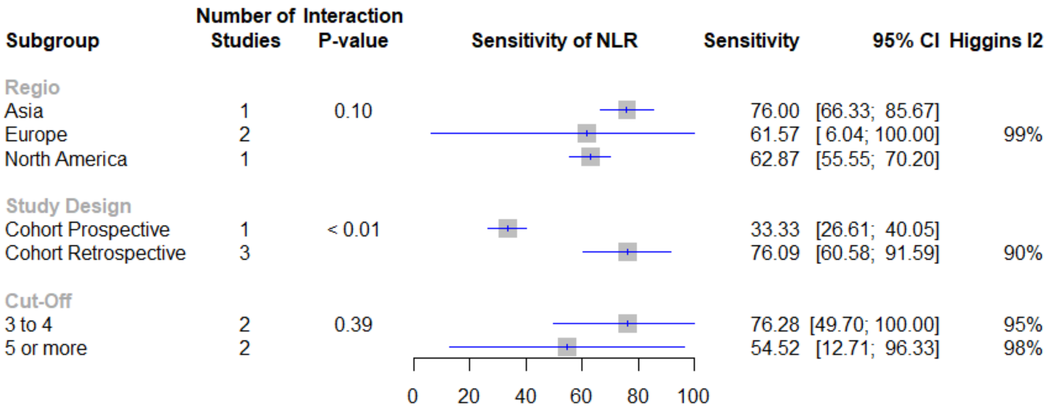

B. Baujat plot of pooled sensitivity in MALE outcome (1-year follow-up)

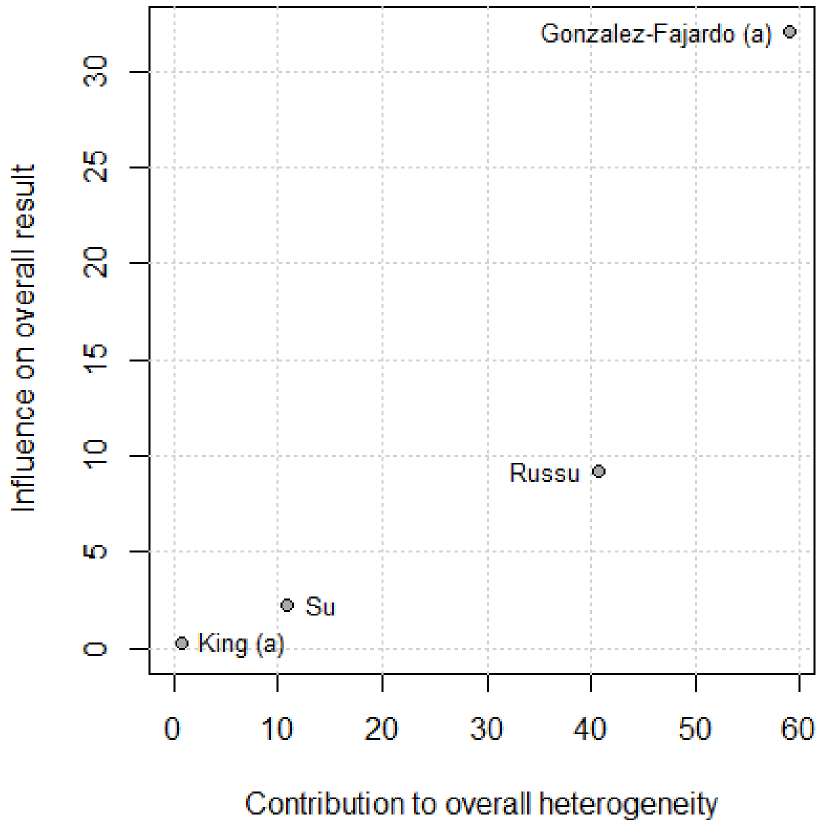

C. Leave-one-out sensitivity analysis of pooled sensitivity in the MALE outcome. Top: 1-year follow-up, Bottom: 3-year follow-up.

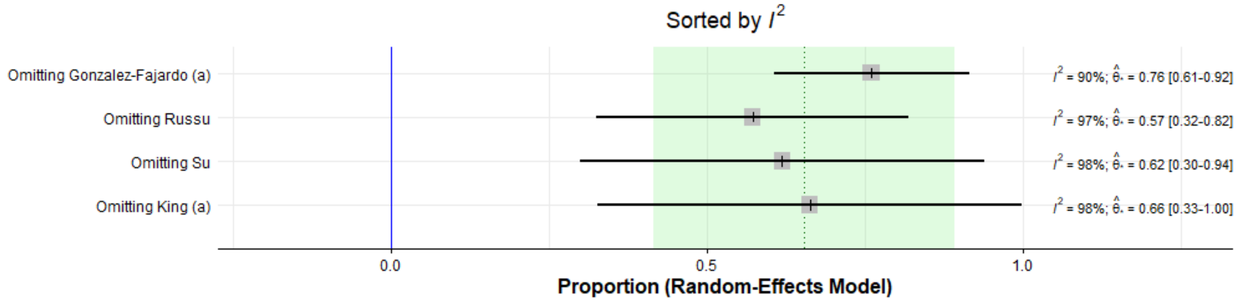

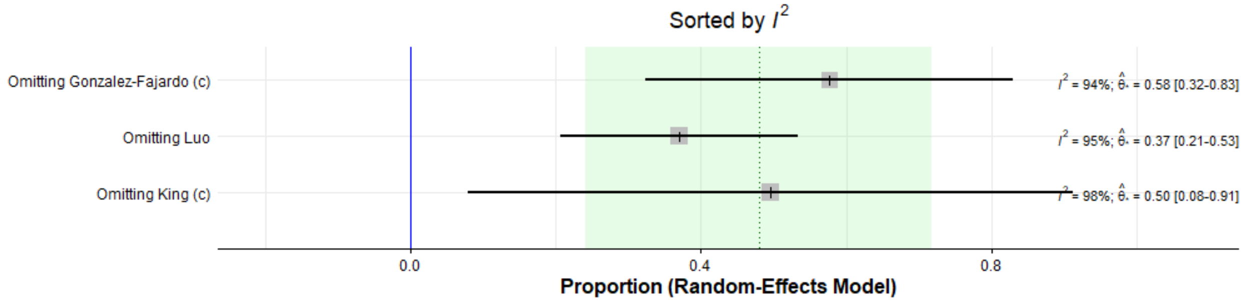

Figure S13. Pooled specificity of high NLR to predict MALE

Study	Ref.	Events	Total	Specificity (%)	95% CI
**Inhospital MALE**					
Bath	[40]	913	2526	36.14	[34.27; 38.05]
**1−year MALE**					
Su	[46]	120	161	74.53	[67.08; 81.06]
Russu	[44]	135	184	73.37	[66.37; 79.60]
Gonzalez−Fajardo (a)	[45]	323	372	86.83	[82.96; 90.09]
King (a)	[41]	239	321	74.45	[69.32; 79.14]
**Random effects model**			**1038**	**77.65**	**[70.98; 84.31]**
Heterogeneity: $I^2 = 89\%$, $\tau^2 = 0.0039$, $p < 0.01$					
**2−year MALE**					
Gonzalez−Fajardo (b)	[45]	287	332	86.45	[82.29; 89.94]
King (b)	[41]	156	197	79.19	[72.84; 84.63]
**Random effects model**			**529**	**83.15**	**[76.07; 90.23]**
Heterogeneity: $I^2 = 77\%$, $\tau^2 = 0.0020$, $p = 0.04$					
**3−year MALE**					
Gonzalez−Fajardo (c)	[45]	265	303	87.46	[83.19; 90.97]
Luo	[58]	81	113	71.68	[62.43; 79.76]
King (c)	[41]	101	122	82.79	[74.90; 89.02]
**Random effects model**			**538**	**81.23**	**[72.29; 90.17]**
Heterogeneity: $I^2 = 83\%$, $\tau^2 = 0.0052$, $p < 0.01$					
**4−year MALE**					
Gonzalez−Fajardo (d)	[45]	242	276	87.68	[83.21; 91.32]
King (d)	[41]	47	57	82.46	[70.09; 91.25]
**Random effects model**			**333**	**86.98**	**[83.37; 90.59]**
Heterogeneity: $I^2 = 0\%$, $\tau^2 = 0$, $p = 0.33$					
**5−year MALE**					
Gonzalez−Fajardo (e)	[45]	225	254	88.58	[84.02; 92.22]

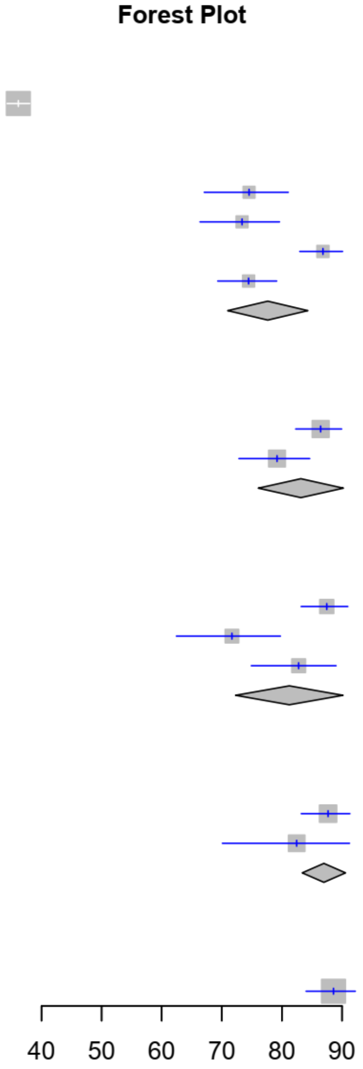

Figure S14. Additional analyses for specificity in MALE outcome

A. Subgroup analysis of pooled specificity in MALE outcome (1-year follow-up)

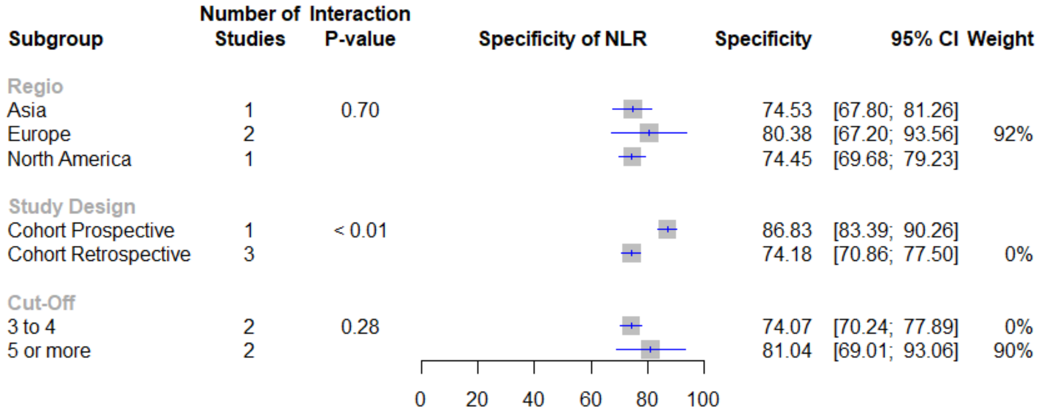

B. Leave-one-out sensitivity analysis of pooled specificity in the MALE outcome. Top: 1-year follow-up, Bottom: 3-year follow-up.

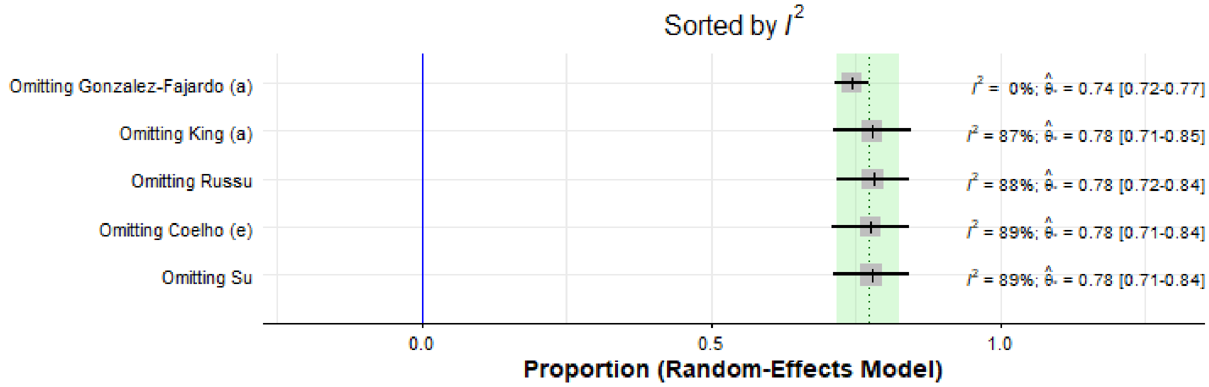

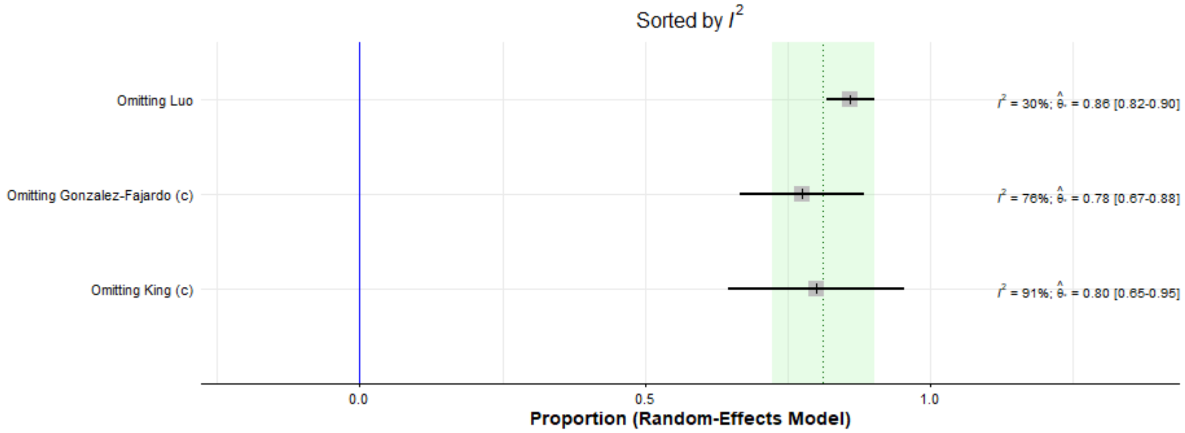

Figure S15. Pooled prevalence of MALE between high and low NLR group

Subgroup	Number of Studies	Interaction P-value	Forest Plot	Prevalence (%)	95% CI	I2
High NLR						
1-year MALE	4	0.03		53.70	[47.33; 60.07]	50%
2-year MALE	2			69.29	[51.41; 87.16]	91%
3-year MALE	3			71.07	[52.18; 89.96]	95%
4-year MALE	2			82.47	[57.97; 100.00]	97%
Low NLR						
1-year MALE	4	< 0.01		16.09	[5.40; 26.78]	97%
2-year MALE	2			42.00	[30.15; 53.84]	91%
3-year MALE	3			41.69	[13.98; 69.39]	98%
4-year MALE	2			65.26	[27.74; 100.00]	99%

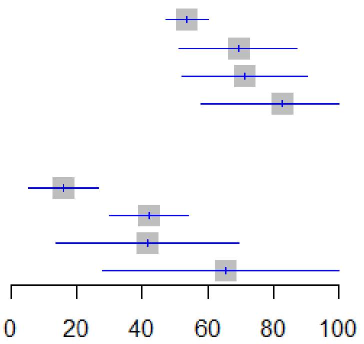

Figure S16. Pooled PPV of high NLR to predict MALE

Study	Ref.	Events	Total	PPV (%)	95% CI
**Inhospital MALE**					
Bath	[40]	149	1762	8.46	[7.20; 9.85]
**1−year MALE**					
Su	[46]	57	98	58.16	[47.77; 68.05]
Russu	[44]	36	85	42.35	[31.70; 53.55]
Gonzalez−Fajardo (a)	[45]	63	112	56.25	[46.56; 65.61]
King (a)	[41]	105	187	56.15	[48.72; 63.38]
**Random effects model**			482	**53.70**	**[47.33; 60.07]**
Heterogeneity: $I^2 = 50\%$, $\tau^2 = 0.0021$, $p = 0.11$					
**2−year MALE**					
Gonzalez−Fajardo (b)	[45]	67	112	59.82	[50.14; 68.97]
King (b)	[41]	146	187	78.07	[71.45; 83.78]
**Random effects model**			299	**69.29**	**[51.41; 87.16]**
Heterogeneity: $I^2 = 91\%$, $\tau^2 = 0.0151$, $p < 0.01$					
**3−year MALE**					
Gonzalez−Fajardo (c)	[45]	74	112	66.07	[56.52; 74.75]
Luo	[58]	42	74	56.76	[44.72; 68.23]
King (c)	[41]	166	187	88.77	[83.35; 92.91]
**Random effects model**			373	**71.07**	**[52.18; 89.96]**
Heterogeneity: $I^2 = 95\%$, $\tau^2 = 0.0260$, $p < 0.01$					
**4−year MALE**					
Gonzalez−Fajardo (d)	[45]	78	112	69.64	[60.24; 77.98]
King (d)	[41]	177	187	94.65	[90.39; 97.41]
**Random effects model**			299	**82.47**	**[57.97; 100.00]**
Heterogeneity: $I^2 = 97\%$, $\tau^2 = 0.0302$, $p < 0.01$					
**5−year MALE**					
Gonzalez−Fajardo (e)	[45]	83	112	74.11	[64.97; 81.92]

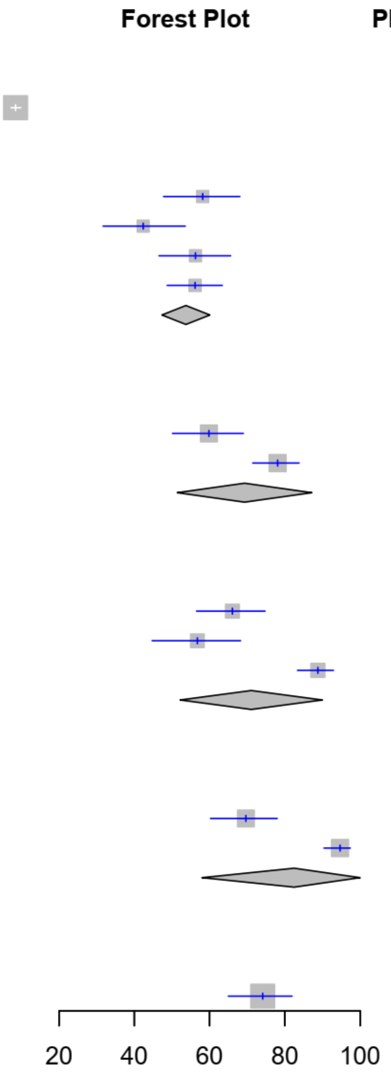

Figure S17. Pooled NPV of high NLR to predict MALE

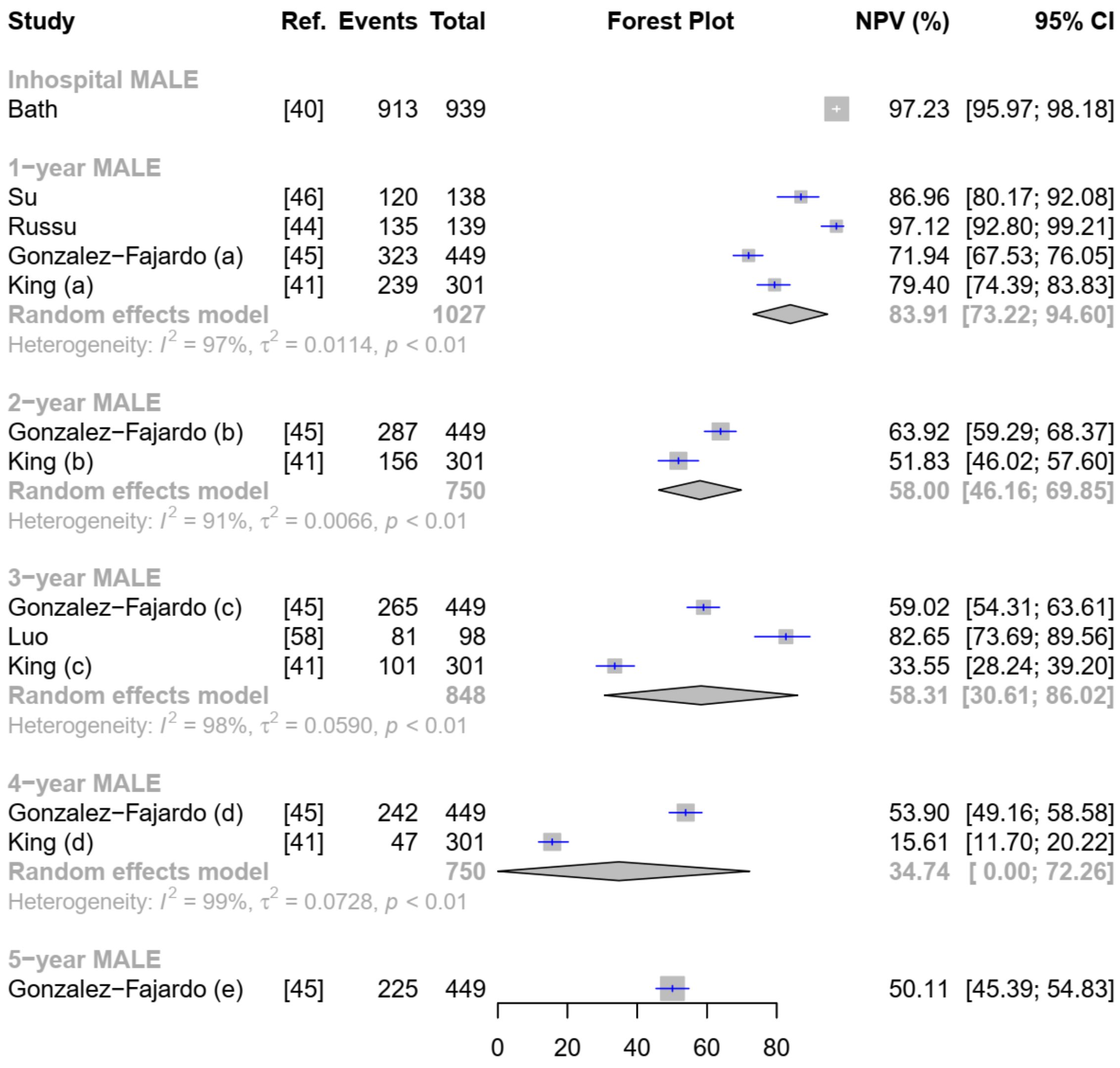

Supplement: sj-pdf-1-vmj-10.1177_1358863X241281699 – Supplemental material for Neutrophil-to-lymphocyte ratio as a prognostic biomarker in patients with peripheral artery disease: A systematic review and meta-analysis [file sj-pdf-1-vmj-10.1177_1358863X241281699.pdf]
